# Supplementary material for: Cleavage of MEP-1 by DPF-3 reveals novel substrate specificity and its impact on reproductive fitness
Source: EMBO Rep. 2026 Jun 19;27(15):4524–43. doi: 10.1038/s44319-026-00844-y (PMC13458333; doi:10.1038/s44319-026-00844-y)
Supplement: Supplementary file 1 [file 44319_2026_844_MOESM1_ESM.pdf]

EMBO reports

Cleavage of MEP-1 by DPF-3 Reveals Novel Substrate Specificity and Its Impact on Reproductive Fitness

Ilkin Aygun, Afzal Amanullah, Jan Seebacher, Daniel Hess, Charlotte Soneson, Ankit Roy, Helge Großhans, and Rajani Kanth Gudipati

Corresponding author(s): Rajani Kanth Gudipati (rajani.gudipatti@amu.edu.pl)

Review Timeline:

Transfer Date:	9th Dec 25
Editorial Decision:	2nd Jan 26
Revision Received:	27th Mar 26
Editorial Decision:	20th May 26
Revision Received:	22nd May 26
Accepted:	10th Jun 26

Editor: Kurt Weir

Transaction Report: This manuscript was transferred to EMBO Reports following peer review at Review Commons.

(Note: With the exception of the correction of typographical or spelling errors that could be a source of ambiguity, letters and reports are not edited. Depending on transfer agreements, referee reports obtained elsewhere may or may not be included in this compilation. Referee reports are anonymous unless the Referee chooses to sign their reports.)

Review #1

1. Evidence, reproducibility and clarity:

Evidence, reproducibility and clarity (Required)

The data is convincing and the conclusions overall are supported by the data. The novelty of the work should be seen in the identification of a new in vivo-relevant substrate of this dipeptidase, and that this new substrate does not carry the typical XP N-terminal signature. The precise role of the processing of MEP-1 remains rather unclear. No effects on MEP-1 interactions were found, and the relevance of MEP-1 stability, or lowered steady-state levels, in relation to its function is not clarified.

A few specific comments:

1. Fig 1: what does the grey mean? These seem to be internal fragments. Is that correct (in other words, noise of the TAIL procedure).
2. Fig 3: it remains unclear from these plots how much of the substrate is converted into product. Does the reaction reach full processing? (same for Fig 2)
3. Figure 4A (Western) needs quantification and a non-tagged control. As presented, the effect of dpf-3 remains rather unclear, and we also do not know what the background is of Ab-signal in extracts not expressing any tag.
4. The enhanced effect of the T3E mutation compared to loss of DPF-3 is explained in terms of redundancies between different DPF enzymes. However, it may just as well be that the T3E allele produces a protein that is inherently less stable (a degron could have been created). This should be discussed as an alternative possibility (that is just as likely as far as I am aware)
5. Fig 5B-E: the images in my PDF file are not of good enough quality to judge the exact defects that may be present.

2. Significance:

Significance (Required)

Aygün et al. report on the identification of novel in vivo substrates of the peptidase DPF-3 in C. elegans/ DPF-3 is a member of the DPPIV family of di-peptidases with clinical relevance, and a better understanding of the activities in vivo is clearly important. Notably, the authors here show that in vivo DPF-3 can cleave following a threonine, an activity that thus far had only been shown in vitro. In particular, they show and validate this for the protein MEP-1. Loss of DPF-3 leads to a loss of steady-state levels of MEP-1, something that was also found in a mep-1 mutant background that produced MEP-1(T3E) protein that is resistant to

DPF-3. This mep-1 allele also recapitulated some of the dpf-3 mutant phenotypes, suggesting that MEP-1 processing, or rather the lack thereof, contributes to the dpf-3 mutant phenotype.

3. How much time do you estimate the authors will need to complete the suggested revisions:

Estimated time to Complete Revisions (Required)

(Decision Recommendation)

Less than 1 month

4. *Review Commons* values the work of reviewers and encourages them to get credit for their work. Select 'Yes' below to register your reviewing activity at Web of Science Reviewer Recognition Service (formerly Publons); note that the content of your review will not be visible on Web of Science.

Web of Science Reviewer Recognition

Yes

Review #2

1. Evidence, reproducibility and clarity:

Evidence, reproducibility and clarity (Required)

Aygun et al. use a mass spectrometry-based assay in C. elegans to identify substrates of the Dipeptidyl Peptidase IV (DPPIV) family member DPF-3. This was motivated by their prior work that showed DPF-3 could cleave after more diverse amino acids than previously thought. After verifying their approach identified genuine DPF-3 targets using an in vitro synthetic peptide and recombinant DPF-3 assay, they focused on MEP-1 which has a non-canonical amino acid cleavage site. They demonstrated that DPF-3 cleavage impacts MEP-1 stability and showed the physiological consequence of disrupting DPF-3 cleavage of MEP-1 is low penetrant reproductive defects and germline defects.

Major Comments:

1. Quantification of western blots with statistics for replicates should be added for Figure

4A.
2. The authors see that the flag::mep-1(T3E) allele shows near complete loss of MEP-1 levels but that MEP-1 levels are not as dramatically changed in dpf-3Δ; flag::mep-1 animals and suggest that this means DPF-3 processing removes a degron signal at the N-terminus of MEP-1 required for the protein stability. Further they argue that the dramatic effect is not observed in dpf-3Δ; flag::mep-1 animals due to the fact that other DPPIV family members could maybe act redundantly to cleave the T3 site in mep-1. However, there currently is no evidence that other DPPIV family members could recognize the T3 site in mep-1 and begs the question of whether the flag tag and T3E mutation elicits some other fate that blocks translation of the mep-1(T3E) mRNA or leads to its degradation. To support that other DPPIV family members act redundantly in the dpf-3Δ; flag::mep-1 animals, the authors could perform the in vitro synthetic peptide assay with other recombinant DPPIV family members to show they are able to cleave MEP-1 in the absence of DPF-3.
3. Why did the authors perform their reproductive assays at 25ºC, which is a heat stress condition? Were the western blots also performed at 25ºC? Does their mep-1(T3E) allele not recapitulate mep-1(q660) phenotypes at 20ºC, which is the standard growth temperature of C. elegans? Would this suggest the role of DPF-3 in regulating MEP-1 is critical for during heat stress and not necessarily during permissive conditions? An explanation for the choice of 25 ºC would be helpful for the reader, and potentially complementary experiments performed at 20 ºC.
4. Does the DPF-3 processing impact localization of MEP-1?
5. OPTIONAL: The authors propose the role of DPF-3 processing is to fine-tune abundance of MEP-1 and it's interaction complexes, if this model is correct, there could be potential gradations in changes of the chromatin landscape between wild-type, dpf-3Δ; flag::mep-1, and flag::mep-1(T3E) animals that reflective the levels of stable MEP-1 protein.

Minor Comments:

1. The fourth paragraph of the results, discussing factors that impact patterns of substrate/product detection was a bit confusing as it is written and where it is placed. The relevance of the different patterns comes later in the MEP-1 results, so it may improve readability to move some of this discussion there.
2. It is unclear why the authors picked GDPH-1 and FAT-1 as candidates to validate the DPF-3 substrates/products found in their screen as these didn't exhibit substrate/product pairs, so some explanation of their choice would be helpful.
3. On Figure 1B, it could be helpful to label the quadrants of the plot as "DPF-3 substrates" and "DPF-3 products".

4. It would be helpful to have a graphic with percentages of reproductive phenotypes for the wild-type and mep-1(T3E) animals in Figure 5.

2. Significance:

Significance (Required)

The work by Aygun et al. is of broad interest and addresses open questions in basic research about post-translational regulation by Dipeptidyl Peptidase IV (DPPIV) family members. The identification of novel substrates and non-canonical cleavage sites is of significance as it challenges current perceptions of DPF-3 targets and advances our understanding of how to study these proteases. Further the authors demonstrate a functional role of DPF-3 in reproduction and through the cleavage of one newly identified target. A strength of the study is the unbiased approach of identifying DPF-3 targets that enabled them to find products cleaved at non-canonical amino acids, but a limitation is the reagents used for lysate preparation; however they acknowledge this. It is another strength that they were able to show a physiological consequence of disrupting the DPF-3 processing of this unexpected target and start to address potential function, although a few additional experiments could bolster their arguments towards this functional role. A limitation in the interpretation of their reproductive assays I see is the choice of growth temperature for the reproductive assays (25ºC) as heat stress can already trigger low penetrance reproductive phenotypes. Further, this entangles their interpretation of the role of DPF-3 processing with its role during stress which could differ from its role during non-stress conditions.

My expertise: epigenetics, reproduction, homeostasis, C. elegans

3. How much time do you estimate the authors will need to complete the suggested revisions:

Estimated time to Complete Revisions (Required)

(Decision Recommendation)

Between 1 and 3 months

4. *Review Commons* values the work of reviewers and encourages them to get credit for their work. Select 'Yes' below to register your reviewing activity at [Web of Science Reviewer Recognition Service](#) (formerly Publons); note that the content of your review will not be visible on Web of Science.

Web of Science Reviewer Recognition

Yes

Review #3

1. Evidence, reproducibility and clarity:

Evidence, reproducibility and clarity (Required)

This is a solid and well-executed study that convincingly identifies DPF-3 as a protease required for MEP-1 processing and stabilization in C. elegans, and explores the molecular and physiological consequences of disrupting this pathway. The combination of biochemical, proteomic, and genetic approaches provides a coherent mechanistic narrative, and multiple lines of evidence support most conclusions. The presentation is generally clear, the data are well described, and the manuscript is carefully organized.

My main concern relates to the IP-mass spectrometry analyses (Fig. 4 and Supp. Fig. 2), which underpin the claim that MEP-1 complex composition is unchanged in the dpf-3 mutant. The p-values for enriched interactors are quite modest, and it is unclear whether this reflects insufficient biological replicates, technical variability, or high background. The authors should clarify the number of replicates, report replicate correlations and missing-value rates, and use a consistent statistical workflow across comparisons. It would also be helpful to state whether the "match-between-runs" method was used. While the conclusion that complex composition remains unchanged may be correct, the data as presented do not rule out the possibility that the apparent similarity arises from limited statistical power. If possible, targeted validation (e.g. co-IP for key partners such as LET-418 or HDA-1) would strengthen this claim, even though co-IPs do not provide a quantitative view

Another point concerns the brood-size assays, which were performed at 25 {degree sign}C. This temperature reduces fertility even in wild-type animals, complicating the interpretation of genotype-specific effects. It would be important to report brood size at 20 {degree sign}C and discuss whether the observed fertility defects are temperature-dependent or intrinsic to the mutations.

Finally, the authors propose that other DPF family members may compensate for loss of dpf-3, based on the more pronounced destabilization of MEP-1(T3E). This is an interesting

idea that could be tested by a targeted RNAi screen or double/triple mutant analysis of other dpf genes in the dpf-3 background.

Overall, the experiments are carefully performed and described, but clarification of the proteomic statistics and additional fertility assays would increase confidence in the robustness of the conclusions.

2. Significance:

Significance (Required)

This study provides an important mechanistic advance in understanding how the protease DPF-3 regulates the stability and function of the chromatin factor MEP-1 in C. elegans. Previous work had implicated DPF-3 in RNA silencing and proteostasis, but its direct substrates and developmental roles were not defined. Using a proteome-wide N-terminomics (TAILS) approach, the authors systematically identify DPF-3 cleavage targets in developing nematodes. This analysis not only recovers canonical DPPIV substrates with Pro or Ala at the P1 position but also uncovers MEP-1 as a noncanonical substrate containing Thr at P1, whose cleavage is essential for its stability and function in germline development.

By integrating quantitative proteomics, mutagenesis, and genetic analysis, the study reveals that loss of DPF-3-dependent cleavage leads to destabilization of MEP-1, defective gonadal migration, and reduced fertility. This discovery broadens the known substrate specificity and biological repertoire of DPF-3, positioning it as a protease that acts on chromatin-associated proteins rather than exclusively on cytosolic or silencing factors. The identification of MEP-1 as a noncanonical target provides a striking example of how proteolytic processing can fine-tune chromatin regulator function in vivo.

The main limitation lies in the modest statistical support for unchanged complex composition in the dpf-3 mutant IP-MS data, which somewhat weakens the conclusion that DPF-3 does not alter MEP-1 complex assembly. Nonetheless, the study presents a coherent and well-supported mechanistic model that significantly extends the functional landscape of DPF-3. The findings will be of broad interest to researchers studying protease biology, chromatin regulation, and germline development, and may have implications for understanding noncanonical DPPIV functions across species.

3. How much time do you estimate the authors will need to complete the suggested revisions:

Estimated time to Complete Revisions (Required)

(Decision Recommendation)

Between 1 and 3 months

4. *Review Commons* values the work of reviewers and encourages them to get credit for their work. Select 'Yes' below to register your reviewing activity at Web of Science Reviewer Recognition Service (formerly Publons); note that the content of your review will not be visible on Web of Science.

Web of Science Reviewer Recognition

Yes

Revision Plan

Manuscript number: RC-2025-03236
Corresponding author(s): Rajani Kanth Gudipati

[The "revision plan" should delineate the revisions that authors intend to carry out in response to the points raised by the referees. It also provides the authors with the opportunity to explain their view of the paper and of the referee reports.

The document is important for the editors of affiliate journals when they make a first decision on the transferred manuscript. It will also be useful to readers of the reprint and help them to obtain a balanced view of the paper.

If you wish to submit a full revision, please use our "Full Revision" template. ***It is important to use the appropriate template to clearly inform the editors of your intentions.****]*

1. General Statements [optional]

This section is optional. Insert here any general statements you wish to make about the goal of the study or about the reviews.

2. Description of the planned revisions

Insert here a point-by-point reply that explains what revisions, additional experimentations and analyses are planned to address the points raised by the referees.

Reviewer #1 (Evidence, reproducibility and clarity (Required)):

The data is convincing and the conclusions overall are supported by the data. The novelty of the work should be seen in the identification of a new in vivo-relevant substrate of this dipeptidase, and that this new substrate does not carry the typical XP N-terminal signature. The precise role of the processing of MEP-1 remains rather unclear. No effects on MEP-1 interactions were found, and the relevance of MEP-1 stability, or lowered steady-state levels, in relation to its function is not clarified.

We thank the reviewer for recognizing the novelty of our work and for their supportive comments. Below, we provide point - by - point responses to the queries raised. We also further clarify the consequences or the lack thereof of MEP-1 processing by the DPF-3 protease.

A few specific comments:
1) Fig 1: what does the grey mean? These seem to be internal fragments. Is that correct (in other words, noise of the TAIL procedure).

Our apologies for not mentioning what the grey dots indicate. The grey dots indicate the peptides whose abundance does not change in *dpf-3* mutant compared to wild-type animals. We will include the below statement in the figure legend of the revised manuscript. "The peptides that do not change significantly are colored grey".

2) Fig 3: it remains unclear from these plots how much of the substrate is converted into product. Does the reaction reach full processing? (same for Fig 2)

We thank the reviewer for raising this point. Depending upon the substrate, a fraction of the substrate is converted into product and the reactions does not reach plateau. For instance, the peptide representing the FAT-1 is turned over much better than the peptide representing MEP-1. We will include the plots quantifying the substrates in the revised manuscript.

3) Figure 4A (Western) needs quantification and a non-tagged control. As presented, the effect of dpf-3 remains rather unclear, and we also do not know what the background is of Ab-signal in extracts not expressing any tag.

To exclude the possibility of background signal, we will repeat the western blot analysis by including lysates from wild-type N2 animals, which do not contain the FLAG epitope. In addition, as noted in our response to comment 4 (below), we will generate and examine additional mutant transgenic lines, and these samples will also be incorporated into the revised western blot. Quantification of all western blot data will be provided. All results related to this point will be included in the revised manuscript.

4) The enhanced effect of the T3E mutation compared to loss of DPF-3 is explained in terms of redundancies between different DPF enzymes. However, it may just as well be that the T3E allele produces a protein that is inherently less stable (a degron could have been created). This should be discussed as an alternative possibility (that is just as likely as far as I am aware)

We thank the reviewer for this valuable comment and fully agree that the enhanced effect of the T3E mutation may result from decreased stability of the mutant protein, potentially due to the creation of a degron. To address this alternative possibility, we will generate and analyse additional mutant transgenic animals (e.g., T3G, T3S) and assess the MEP-1 protein levels. Furthermore, we will assess MEP-1 protein levels in *dpf-3; dpf-4* and *dpf-3; dpf-5* double mutant animals. We selected *dpf-4* and *dpf-5* because we have observed synthetic sterility in these double mutants (unpublished data).

In addition to evaluating MEP-1 protein levels by western blot and providing full quantitative details, we will also carefully examine these animals for morphological defects.

5) Fig 5B-E: the images in my PDF file are not of good enough quality to judge the exact defects that may be present.

We apologize for the inconvenience caused by the low image quality in the submitted PDF. We will provide all images in high resolution to allow clear assessment of the observed phenotypes.

Reviewer #1 (Significance (Required)):

Aygün et al. report on the identification of novel in vivo substrates of the peptidase DPF-3 in C. elegans/ DPF-3 is a member of the DPPIV family of di-peptidases with clinical relevance, and a better understanding of the activities in vivo is clearly important. Notably, the authors here show that in vivo DPF-3 can cleave following a threonine, an activity that thus far had only been shown in vitro. In particular, they show and validate this for the protein MEP-1. Loss of DPF-3 leads to a loss of steady-state levels of MEP-1, something that was also found in a mep-1 mutant background that produced MEP-1(T3E) protein that is resistant to DPF-3. This mep-1 allele also recapitulated some of the dpf-3 mutant phenotypes, suggesting that MEP-1 processing, or rather the lack thereof, contributes to the dpf-3 mutant phenotype.

Once again, we thank the reviewer for critically evaluating our manuscript, recognizing the novelty of our work, and providing constructive comments that will help us further improve the manuscript.

Reviewer #2 (Evidence, reproducibility and clarity (Required)):

Aygun et al. use a mass spectrometry-based assay in C. elegans to identify substrates of the Dipeptidyl Peptidase IV (DPPIV) family member DPF-3. This was motivated by their prior work that showed DPF-3 could cleave after more diverse amino acids than previously thought. After verifying their approach identified genuine DPF-3 targets using an in vitro synthetic peptide and recombinant DPF-3 assay, they focused on MEP-1 which has a non-canonical amino acid cleavage site. They demonstrated that DPF-3 cleavage impacts MEP-1 stability and showed the physiological consequence of disrupting DPF-3 cleavage of MEP-1 is low penetrant reproductive defects and germline defects.

Major Comments:

1. Quantification of western blots with statistics for replicates should be added for Figure 4A.

We apologize for the omission of quantification in Figure 4A. In the revised version, we will include quantitative analysis of the western blots with statistical evaluation of biological

replicates. In addition, as suggested by Reviewer #1 (comment 3), we will include additional samples to rule out background signal and will provide a new, fully quantified western blot.

2. The authors see that the flag::mep-1(T3E) allele shows near complete loss of MEP-1 levels but that MEP-1 levels are not as dramatically changed in dpf-3∆; flag::mep-1 animals and suggest that this means DPF-3 processing removes a degron signal at the N-terminus of MEP-1 required for the protein stability. Further they argue that the dramatic effect is not observed in dpf-3∆; flag::mep-1 animals due to the fact that other DPPIV family members could maybe act redundantly to cleave the T3 site in mep-1. However, there currently is no evidence that other DPPIV family members could recognize the T3 site in mep-1 and begs the question of whether the flag tag and T3E mutation elicits some other fate that blocks translation of the mep-1(T3E) mRNA or leads to its degradation. To support that other DPPIV family members act redundantly in the dpf-3∆; flag::mep-1 animals, the authors could perform the in vitro synthetic peptide assay with other recombinant DPPIV family members to show they are able to cleave MEP-1 in the absence of DPF-3.

We appreciate the reviewer's insightful comments. The reviewer raises a valid concern regarding whether the T3E mutation itself could lead to degradation of the MEP-1 protein. This issue was also noted by Reviewer #1 (comment 4), and we will address it by generating and analysing additional *flag::mep-1* mutant transgenic lines, such as T3G and T3S, to determine whether the observed reduction in MEP-1 levels is specific to the T3E substitution.

Regarding the possibility that other DPPIV family members may act redundantly to process MEP-1, we agree that direct biochemical evidence would be ideal. The *C. elegans* DPPIV family consists of six predicted catalytically active members (based on conservation of the catalytic triad) and one potentially inactive paralog. However, these proteases are not commercially available, and to date, we have only successfully produced recombinant DPF-3 in insect cells, a process that required more than two years of optimization due to severe expression and aggregation issues. Producing additional recombinant family members within a reasonable timeframe would therefore be technically challenging.

As an alternative approach, we propose to test our hypothesis genetically by examining MEP-1 levels in *dpf-4* or *dpf-5* single mutants, as well as in double or triple mutant backgrounds. We selected *dpf-4* and *dpf-5* because we have observed synthetic sterility in these double mutants (unpublished data). This will allow us to assess whether other DPPIV family members contribute redundantly to MEP-1 processing in vivo.

3. Why did the authors perform their reproductive assays at 25ºC, which is a heat stress condition? Were the western blots also performed at 25ºC? Does their mep-1(T3E) allele not recapitulate mep-1(q660) phenotypes at 20ºC, which is the standard growth temperature of C. elegans? Would this suggest the role of DPF-3 in regulating MEP-1 is critical for during heat

stress and not necessarily during permissive conditions? An explanation for the choice of 25 ºC would be helpful for the reader, and potentially complementary experiments performed at 20 ºC.

We thank the reviewer for this important comment. All experiments in this study, including the TAILS-based substrate identification and western blot analyses, were performed using animals cultured at 25℃. We chose this temperature for consistency, as the TAILS samples were prepared from animals grown at 25℃, and all subsequent experiments were conducted under the same conditions to ensure comparability.

We agree that examining these phenotypes at 20℃, the standard growth temperature for *C. elegans*, could provide valuable additional insight. As suggested, we repeated the relevant experiments at 20℃ to determine whether the observed effects are specific to heat stress conditions or reflect a more general role of DPF-3 in regulating MEP-1 stability and its function. As shown below, mutant animals showed a significant reduction in brood size at 20℃, demonstrating that the effect of DPF-3 on MEP-1 stability and function is not temperature dependent and reflects a broader role for DPF-3 in reproductive fitness. We will incorporate the below figure in the revised manuscript.

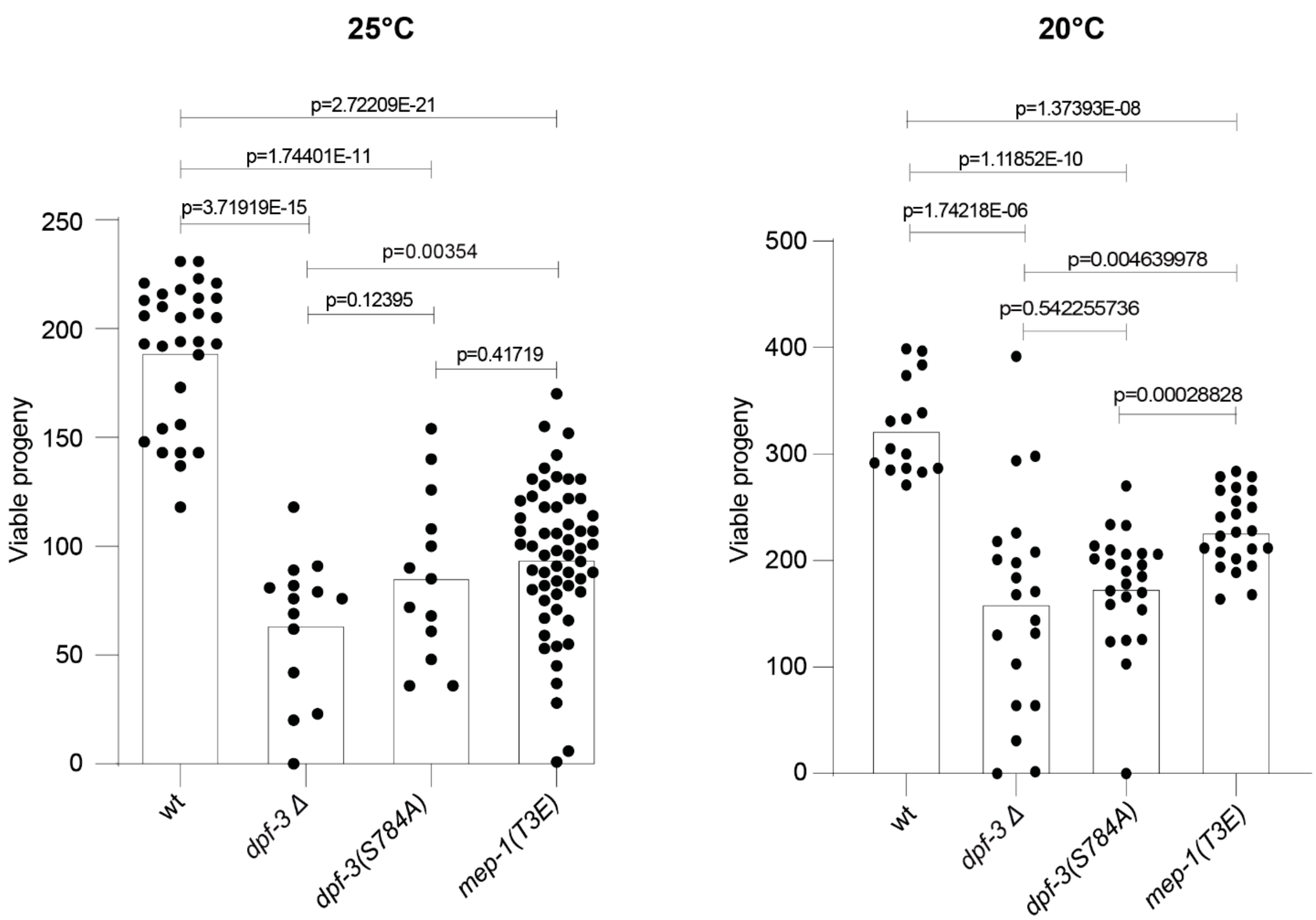

4. Does the DPF-3 processing impact localization of MEP-1?

We thank the reviewer for raising this point, which we were also questioning. We have not yet examined whether DPF-3 mediated processing affects MEP-1 localization. We will address this by generating transgenic animals expressing GFP::MEP-1 and assessing its localization in the presence and absence of dpf-3.

5. OPTIONAL: The authors propose the role of DPF-3 processing is to fine-tune abundance of MEP-1 and it's interaction complexes, if this model is correct, there could be potential gradations in changes of the chromatin landscape between wild-type, dpf-3Δ; flag::mep-1, and flag::mep-1(T3E) animals that reflective the levels of stable MEP-1 protein.

We thank the reviewer for this insightful suggestion. Assessing potential changes in the chromatin landscape among wild-type, *dpf-3Δ; flag::mep-1*, and *flag::mep-1(T3E)* animals could indeed provide valuable information about the impact of DPF-3 mediated processing on MEP-1 function and its associated complexes. However, such experiments would represent a major undertaking, requiring genome-wide chromatin analyses, and we believe that this is beyond the scope of the current manuscript.

Minor Comments:
1. The fourth paragraph of the results, discussing factors that impact patterns of substrate/product detection was a bit confusing as it is written and where it is placed. The relevance of the different patterns comes later in the MEP-1 results, so it may improve readability to move some of this discussion there.

We thank the reviewer for this helpful suggestion and will revise the text accordingly to incorporate the recommended changes in the revised manuscript.

2. It is unclear why the authors picked GDPH-1 and FAT-1 as candidates to validate the DPF-3 substrates/products found in their screen as these didn't exhibit substrate/product pairs, so some explanation of their choice would be helpful.

Our goal was to validate the TAILS results using an orthogonal approach - an in vitro assay with synthetic peptides representing the N-termini of the identified substrates. We selected GDPH-1 and FAT-1 as representative examples because these peptides contained Pro and Ala residues respectively, at the P1 position. These candidates were chosen randomly from the list of putative substrates for initial validation. We will include this information in the revised manuscript.

3. On Figure 1B, it could be helpful to label the quadrants of the plot as "DPF-3 substrates" and "DPF-3 products".

Thank you for the suggestion and we will include it in the revised manuscript.

4. It would be helpful to have a graphic with percentages of reproductive phenotypes for the wild-type and mep-1(T3E) animals in Figure 5.

We thank the reviewer for this suggestion and will include the suggested graph in the revised manuscript.

Reviewer #2 (Significance (Required)):

The work by Aygun et al. is of broad interest and addresses open questions in basic research about post-translational regulation by Dipeptidyl Peptidase IV (DPPIV) family members. The identification of novel substrates and non-canonical cleavage sites is of significance as it challenges current perceptions of DPF-3 targets and advances our understanding of how to study these proteases. Further the authors demonstrate a functional role of DPF-3 in reproduction and through the cleavage of one newly identified target. A strength of the study is the unbiased approach of identifying DPF-3 targets that enabled them to find products cleaved at non-canonical amino acids, but a limitation is the reagents used for lysate preparation; however they acknowledge this. It is another strength that they were able to show a physiological consequence of disrupting the DPF-3 processing of this unexpected target and start to address potential function, although a few additional experiments could bolster their arguments towards this functional role. A limitation in the interpretation of their reproductive assays I see is the choice of growth temperature for the reproductive assays (25ºC) as heat stress can already trigger low penetrance reproductive phenotypes. Further, this entangles their interpretation of the role of DPF-3 processing with its role during stress which could differ from its role during non-stress conditions. My expertise: epigenetics, reproduction, homeostasis, C. elegans

We sincerely thank the reviewer for the thoughtful and constructive evaluation of our work. We appreciate their recognition of the novelty and significance of identifying non-canonical DPF-3 cleavage sites and newly uncovered substrates, as well as the importance of our unbiased proteomic approach. We are also grateful for the reviewer's acknowledgement of the physiological relevance and broad interest of our findings and the strengths of our functional analyses.

Reviewer #3 (Evidence, reproducibility and clarity (Required)):

This is a solid and well-executed study that convincingly identifies DPF-3 as a protease required for MEP-1 processing and stabilization in C. elegans, and explores the molecular and physiological consequences of disrupting this pathway. The combination of biochemical, proteomic, and genetic approaches provides a coherent mechanistic narrative, and multiple lines of evidence support most conclusions. The presentation is generally clear, the data are well described, and the manuscript is carefully organized.

Revision Plan

We sincerely thank the reviewer for their thoughtful evaluation of our work. We are grateful for the positive and encouraging feedback regarding the clarity, organization, and overall quality of our study. We particularly appreciate the reviewer's recognition of our integrated biochemical, proteomic, and genetic approaches, and their acknowledgment that these complementary lines of evidence establish a coherent mechanistic framework for DPF-3 mediated processing and stabilization of MEP-1. We are pleased that the strength of the data and the significance of the physiological implications came through clearly. Thank you again for your constructive and supportive assessment of our manuscript.

1. My main concern relates to the IP-mass spectrometry analyses (Fig. 4 and Supp. Fig. 2), which underpin the claim that MEP-1 complex composition is unchanged in the dpf-3 mutant. The p-values for enriched interactors are quite modest, and it is unclear whether this reflects insufficient biological replicates, technical variability, or high background. The authors should clarify the number of replicates, report replicate correlations and missing-value rates, and use a consistent statistical workflow across comparisons. It would also be helpful to state whether the "match-between-runs" method was used. While the conclusion that complex composition remains unchanged may be correct, the data as presented do not rule out the possibility that the apparent similarity arises from limited statistical power. If possible, targeted validation (e.g. co-IP for key partners such as LET-418 or HDA-1) would strengthen this claim, even though co-IPs do not provide a quantitative view

We thank the reviewer for this careful and constructive assessment of our IP - mass spectrometry analyses. We appreciate the reviewer's point that the modest p - values warrant clarification, and we agree that additional details regarding our statistical approach will strengthen the manuscript. In the revised version, we will re-analyze the data using a consistent statistical workflow across all comparisons and will provide complete information on the number of biological replicates, replicate correlations, and missing-value rates. We will also explicitly state whether the "match-between-runs" feature was used.

We performed at least three biological replicates for each condition, and we will clearly include this information in the updated manuscript. We agree that targeted validation of key interactors such as LET-418 or HDA-1 would further support our conclusions. While these assays are not quantitative, we are willing to perform them as an additional line of evidence. Given the presence of biological triplicates, we anticipate that the use of appropriate statistical methods will offer a more quantitatively robust evaluation of complex composition, which will also be reflected in the revised manuscript.

2. Another point concerns the brood-size assays, which were performed at 25 {degree sign}C. This temperature reduces fertility even in wild-type animals, complicating the interpretation of genotype-specific effects. It would be important to report brood size at 20 {degree sign}C and discuss whether the observed fertility defects are temperature-dependent or intrinsic to the mutations.

We thank the reviewer for this valuable comment, which was also raised by reviewer #2 (comment 3). We agree that examining brood size at 20℃ would help clarify whether the observed fertility defects are temperature dependent or intrinsic to the mutations. We quantified the brood size at 20℃. As shown below, mutant animals showed a significant reduction in brood size at 20℃, demonstrating that the effect of DPF-3 on MEP-1 stability and function is not temperature dependent and reflects a broader role for DPF-3 in reproductive fitness. We will incorporate the below figure in the revised manuscript.

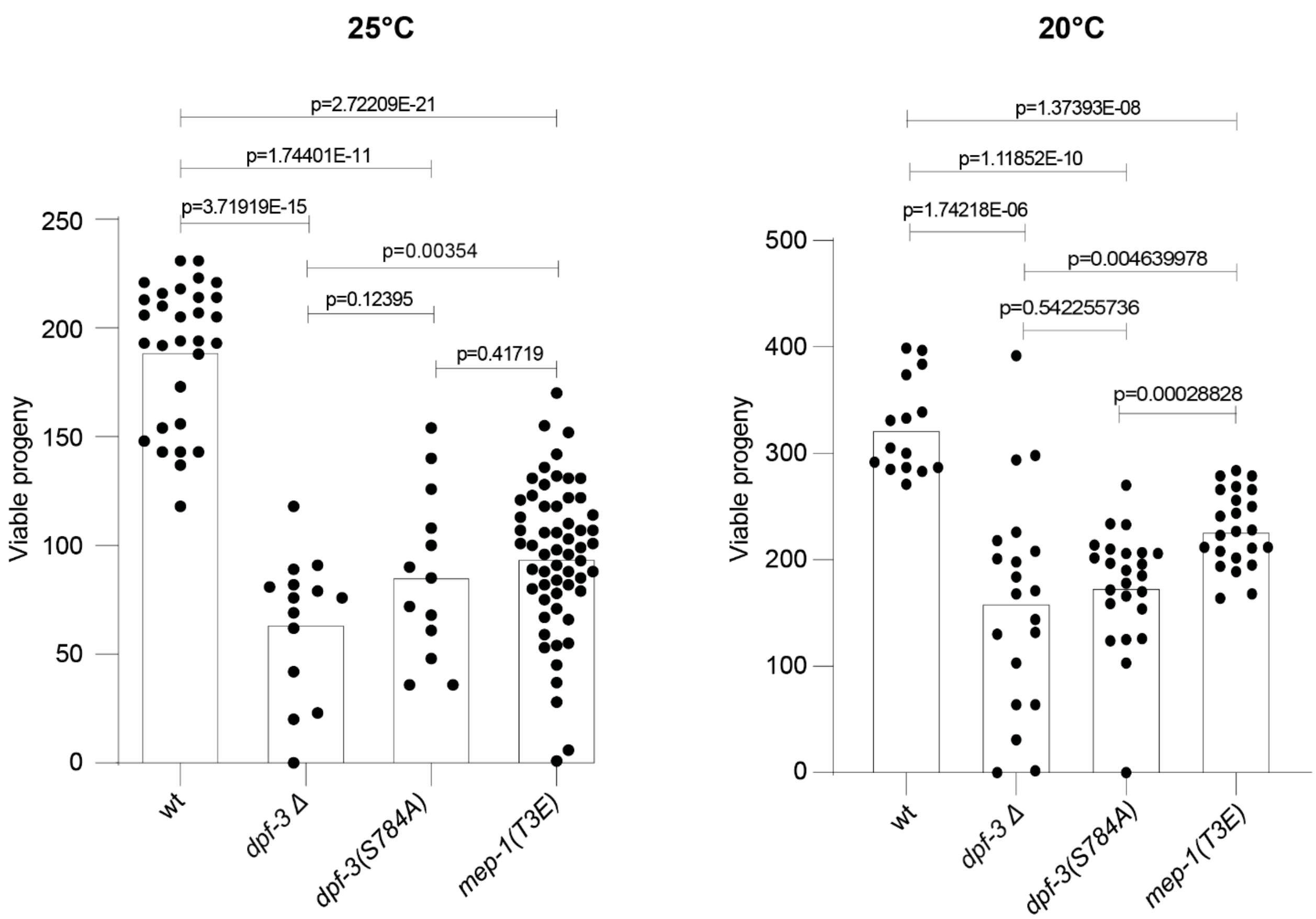

3. Finally, the authors propose that other DPF family members may compensate for loss of dpf-3, based on the more pronounced destabilization of MEP-1(T3E). This is an interesting idea that could be tested by a targeted RNAi screen or double/triple mutant analysis of other dpf genes in the dpf-3 background.

We thank the reviewer for this insightful comment, which is also in line with comment 2 from reviewer #2. We plan to test this hypothesis genetically by examining MEP-1 levels in *dpf-4* and *dpf-5* single mutants, as well as in double or triple mutant backgrounds with dpf-3. We selected

dpf-4 and *dpf-5* because we have observed synthetic sterility in these double mutants (unpublished data). This approach will allow us to determine whether other DPPIV family members act redundantly to mediate MEP-1 processing *in vivo*.

4. Overall, the experiments are carefully performed and described, but clarification of the proteomic statistics and additional fertility assays would increase confidence in the robustness of the conclusions.

We thank the reviewer for the positive feedback. We will include the relevant statistical analyses for the proteomics data (as outlined above in response to comment 1). We have performed additional fertility assays as suggested at 20℃ that strengthens the robustness of our conclusions.

Reviewer #3 (Significance (Required)):

This study provides an important mechanistic advance in understanding how the protease DPF-3 regulates the stability and function of the chromatin factor MEP-1 in C. elegans. Previous work had implicated DPF-3 in RNA silencing and proteostasis, but its direct substrates and developmental roles were not defined. Using a proteome-wide N-terminomics (TAILS) approach, the authors systematically identify DPF-3 cleavage targets in developing nematodes. This analysis not only recovers canonical DPPIV substrates with Pro or Ala at the P1 position but also uncovers MEP-1 as a noncanonical substrate containing Thr at P1, whose cleavage is essential for its stability and function in germline development.

By integrating quantitative proteomics, mutagenesis, and genetic analysis, the study reveals that loss of DPF-3-dependent cleavage leads to destabilization of MEP-1, defective gonadal migration, and reduced fertility. This discovery broadens the known substrate specificity and biological repertoire of DPF-3, positioning it as a protease that acts on chromatin-associated proteins rather than exclusively on cytosolic or silencing factors. The identification of MEP-1 as a noncanonical target provides a striking example of how proteolytic processing can fine-tune chromatin regulator function in vivo.

The main limitation lies in the modest statistical support for unchanged complex composition in the dpf-3 mutant IP-MS data, which somewhat weakens the conclusion that DPF-3 does not alter MEP-1 complex assembly. Nonetheless, the study presents a coherent and well-supported mechanistic model that significantly extends the functional landscape of DPF-3. The findings will be of broad interest to researchers studying protease biology, chromatin regulation, and germline development, and may have implications for understanding noncanonical DPPIV functions across species.

We thank the reviewer for broadly endorsing our manuscript and for highlighting the significance of our findings. We appreciate the comment regarding the statistical analysis of the MEP-1 protein complex. We will provide a more detailed analysis of the immunoprecipitation - mass

spectrometry (IP - MS) datasets, including appropriate statistical evaluation, to strengthen the support for our conclusions.

3. Description of the revisions that have already been incorporated in the transferred manuscript

Please insert a point-by-point reply describing the revisions that were already carried out and included in the transferred manuscript. If no revisions have been carried out yet, please leave this section empty.

4. Description of analyses that authors prefer not to carry out

Please include a point-by-point response explaining why some of the requested data or additional analyses might not be necessary or cannot be provided within the scope of a revision. This can be due to time or resource limitations or in case of disagreement about the necessity of such additional data given the scope of the study. Please leave empty if not applicable.

1. Reviewer #2, in comment #2, suggested testing additional recombinant DPPIV family proteases *in vitro* using the synthetic MEP-1 peptide to determine whether they can cleave this substrate. We would like to note that recombinant *C. elegans* DPPIV family proteases are not commercially available. The production of DPF-3 alone, one of the six potentially active *C. elegans* DPFs, required more than two years of intensive optimization due to severe expression and aggregation challenges. Therefore, generating additional recombinant family members is extremely challenging within a reasonable timeframe.

Instead, we propose an alternative genetic approach that directly tests whether other DPFs influence endogenous MEP-1 protein levels in vivo. Specifically, we will analyze MEP-1 abundance in *dpf-4* and *dpf-5* knockout backgrounds for the reasons described earlier in this document (Reviewer #2, comment #2 and the 3rd point of Reviewer #3). This experiment will allow us to assess potential functional redundancy or compensatory processing of MEP-1 by other DPF family members under physiological conditions.

2. Reviewer #2, in comment #5, (noted as optional) proposes examining whether changes in the chromatin landscape across different genotypes might reflect altered MEP-1 protein levels. We agree that assessing genome-wide chromatin profiles in wild-type, *dpf-3Δ; flag::mep-1*, and *flag::mep-1(T3E)* animals could yield valuable insights into how DPF-3 mediated processing influences MEP-1 function and its associated complexes. However, such analyses would constitute a major undertaking, requiring comprehensive and technically demanding chromatin-level assays. We therefore believe that this work is beyond the scope of the current manuscript.

Dear Dr. Gudipati

Thank you for the transfer of your research manuscript to our journal. I have now read and discussed your work with my colleagues here and assessed the accompanying referee reports from Review Commons.

The Review Commons referees express interest in the work's finding of a noncanonical role for a Dipeptidyl Peptidase IV (DPPIV) family member.

Given this support and given that you plan to address all referee concerns and to further strengthen your data, we would like to invite you to revise your manuscript in line with your submitted revision plan. Please address all referee concerns in a complete point-by-point response. Acceptance of the manuscript will depend on a positive outcome of a second round of review. It is EMBO Reports policy to allow a single round of revision only and acceptance or rejection of the manuscript will therefore depend on the completeness of your responses included in the next, final version of the manuscript.

We realize that it is difficult to revise to a specific deadline. In the interest of protecting the conceptual advance provided by the work, we recommend a revision within 3 months (April 2nd). Please discuss the revision progress ahead of this time with the editor if you require more time to complete the revisions.

I am also happy to discuss the revision further via e-mail or a video call, if you wish.

You can either publish the study as a short report or as a full article. For short reports, the revised manuscript should not exceed 27,000 characters (including spaces but excluding materials & methods and references) and 5 main plus 5 expanded view figures. The results and discussion sections must further be combined, which will help to shorten the manuscript text by eliminating some redundancy that is inevitable when discussing the same experiments twice. For a normal article there are no length limitations, but it should have more than 5 main figures and the results and discussion sections must be separate. In both cases, the entire materials and methods must be included in the main manuscript file.

================
IMPORTANT NOTE:

We perform an initial quality control of all revised manuscripts before re-review. Your manuscript will FAIL this control and the handling will be delayed IN CASE the following APPLIES:

1) A data availability section providing access to data deposited in public databases is missing. If you have not deposited any data, please add a sentence to the data availability section that explains that.

2) Your manuscript contains statistics and error bars based on n=2. Please use scatter blots in these cases. No statistics should be calculated if n=2.

When submitting your revised manuscript, please carefully review the instructions that follow below. Failure to include requested items will delay the evaluation of your revision.

================

When submitting your revised manuscript, we will require:

1) a .docx formatted version of the manuscript text (including legends for main figures, EV figures and tables). Please make sure that the changes are highlighted to be clearly visible.

2) individual production quality figure files as .eps, .tif, .jpg (one file per figure).
Please download our Figure Preparation Guidelines (figure preparation pdf) from our Author Guidelines pages https://www.embopress.org/page/journal/14693178/authorguide for more info on how to prepare your figures.

3) a .docx formatted letter INCLUDING the reviewers' reports and your detailed point-by-point responses to their comments. As part of the EMBO Press transparent editorial process, the point-by-point response is part of the Review Process File (RPF), which will be published alongside your paper.

4) a complete author checklist, which you can download from our author guidelines (). Please insert information in the checklist that is also reflected in the manuscript. The completed author checklist will also be part of the RPF.

5) Please note that all corresponding authors are required to supply an ORCID ID for their name upon submission of a revised

manuscript (). Please find instructions on how to link your ORCID ID to your account in our manuscript tracking system in our Author guidelines
()

6) We replaced Supplementary Information with Expanded View (EV) Figures and Tables that are collapsible/expandable online. A maximum of 5 EV Figures can be typeset. EV Figures should be cited as 'Figure EV1, Figure EV2" etc... in the text and their respective legends should be included in the main text after the legends of regular figures.

- For the figures that you do NOT wish to display as Expanded View figures, they should be bundled together with their legends in a single PDF file called *Appendix*, which should start with a short Table of Content. Appendix figures should be referred to in the main text as: "Appendix Figure S1, Appendix Figure S2" etc. See detailed instructions regarding expanded view here:

- Additional Tables/Datasets should be labeled and referred to as Table EV1, Dataset EV1, etc. Legends have to be provided in a separate tab in case of .xls files. Alternatively, the legend can be supplied as a separate text file (README) and zipped together with the Table/Dataset file.

7) Please note that a Data Availability section at the end of Materials and Methods is now mandatory. In case you have no data that requires deposition in a public database, please state so instead of refereeing to the database.
See also < https://www.embopress.org/page/journal/14693178/authorguide#dataavailability>). Please note that the Data Availability Section is restricted to new primary data that are part of this study.

8) At EMBO Press we ask authors to provide source data for the main manuscript figures. You will receive a separate email with instructions for providing source data with your revised manuscript, including how to upload and organize the files.

Additional information on source data and instruction on how to label the files are available

9) The journal requires a statement specifying whether or not authors have competing interests (defined as all potential or actual interests that could be perceived to influence the presentation or interpretation of an article). In case of competing interests, this must be specified in your disclosure statement. Further information: https://www.embopress.org/competing-interests

10) Figure legends and data quantification:
The following points must be specified in each figure legend:

- the name of the statistical test used to generate error bars and P values,
- the EXACT p-values,
- the number (n) of independent experiments (please specify technical or biological replicates) underlying each data point,
- the nature of the bars and error bars (s.d., s.e.m.)

- If the data are obtained from n {less than or equal to} 5, show the individual data points in addition to the SD or SEM.
- If the data are obtained from n {less than or equal to} 2, use scatter blots showing the individual data points.

Discussion of statistical methodology can be reported in the materials and methods section, but figure legends should contain a basic description of n, P and the test applied.

See also the guidelines for figure legend preparation:
https://www.embopress.org/page/journal/14693178/authorguide#figureformat

- Please also include scale bars in all microscopy images and define their size in the legend, not the image.

11) Our journal encourages inclusion of *data citations in the reference list* to directly cite datasets that were re-used and obtained from public databases. Data citations in the article text are distinct from normal bibliographical citations and should directly link to the database records from which the data can be accessed. In the main text, data citations are formatted as follows: "Data ref: Smith et al, 2001" or "Data ref: NCBI Sequence Read Archive PRJNA342805, 2017". In the Reference list, data citations must be labeled with "[DATASET]". A data reference must provide the database name, accession number/identifiers and a resolvable link to the landing page from which the data can be accessed at the end of the reference. Further instructions are available at .

12) All Materials and Methods need to be described in the main text using our 'Structured Methods' format. According to this format, the Methods section includes a Reagents and Tools Table (listing key reagents, experimental models, software and relevant equipment and including their sources and relevant identifiers) followed by a Methods and Protocols section describing the methods, ideally using a step-by-step protocol format. The aim is to facilitate adoption of the methodologies across labs. Please download and fill our Reagents and Tools Table template (.docx), which you can find in our author guidelines: https://www.embopress.org/page/journal/14693178/authorguide#structuredmethods.

When submitting your revised manuscript, please do not include the Reagents and Tools Table in the Methods section of the manuscript but upload it as a separate file choosing the file type "Reagent Table".
An example of a Method paper with Structured Methods can be found here:
https://www.embopress.org/doi/10.15252/msb.20178071.

13) As part of the EMBO publication's Transparent Editorial Process, EMBO Reports publishes online a Review Process File to accompany accepted manuscripts. This File will be published in conjunction with your paper and will include the referee reports, your point-by-point response and all pertinent correspondence relating to the manuscript.

You are able to opt out of this by letting the editorial office know (emboreports@embo.org). If you do opt out, the Review Process File link will point to the following statement: "No Review Process File is available with this article, as the authors have chosen not to make the review process public in this case."

We would also welcome the submission of cover suggestions, or motifs to be used by our Graphics Illustrator in designing a cover.

I look forward to seeing a revised form of your manuscript when it is ready and please let me know if you have questions or comments regarding the revision.

Yours sincerely,

Kurt Weir
Editor
EMBO Reports

Reviewer #1 (Evidence, reproducibility and clarity (Required)):

The data is convincing and the conclusions overall are supported by the data. The novelty of the work should be seen in the identification of a new in vivo-relevant substrate of this dipeptidase, and that this new substrate does not carry the typical XP N-terminal signature. The precise role of the processing of MEP-1 remains rather unclear. No effects on MEP-1 interactions were found, and the relevance of MEP-1 stability, or lowered steady-state levels, in relation to its function is not clarified.

We thank the reviewer for recognizing the novelty of our work and for their supportive comments. Below, we provide point - by - point responses to the queries raised. We also further clarify the consequences or the lack thereof of MEP-1 processing by the DPF-3 protease.

A few specific comments:
1) Fig 1: what does the grey mean? These seem to be internal fragments. Is that correct (in other words, noise of the TAIL procedure).

Our apologies for not mentioning what the grey dots indicate. The grey dots indicate the peptides whose abundance does not change in *dpf-3* mutant compared to wild-type animals. We included the below statement in the figure legend of the revised manuscript.
“All other peptides are colored gray”.

2) Fig 3: it remains unclear from these plots how much of the substrate is converted into product. Does the reaction reach full processing? (same for Fig 2)

We thank the reviewer for raising this point. The extent of substrate conversion depends on the substrate, and under the conditions tested, the reaction does not always reach a plateau. This reflects differences in how efficiently each substrate is recognized and processed by the enzyme. For instance, the peptide representing the FAT-1 is turned over much better than the peptide representing MEP-1. We included the plot quantifying the FAT-1 substrate in Fig.2C in the revised manuscript.

3) Figure 4A (Western) needs quantification and a non-tagged control. As presented, the effect of dpf-3 remains rather unclear, and we also do not know what the background is of Ab-signal in extracts not expressing any tag.

To exclude the possibility of background signal, we repeated the western blot analysis by including lysates from wild-type N2 animals, which do not contain the FLAG epitope. Additionally, as noted in our response to comment 4 (below), we generated additional mutant transgenic lines, and quantified the MEP-1 levels by western blot. Quantification of all western blot data is also included. The data is included in Fig. 4A,B.

4) The enhanced effect of the T3E mutation compared to loss of DPF-3 is explained in terms of redundancies between different DPF enzymes. However, it may just as well be that the T3E allele produces a protein that is inherently less stable (a degron could have been

created). This should be discussed as an alternative possibility (that is just as likely as far as I am aware)

We thank the reviewer for this valuable comment and fully agree that the enhanced effect of the T3E mutation may result from decreased stability of the mutant protein, potentially due to the creation of a degron. To address this alternative possibility, we generated the following additional mutant transgenic animals: *mep-1(T3G), mep-1(T3M), mep-1(T3L)* and *mep-1(T3I)* and assessed the MEP-1 protein levels (Fig. 4A). Interestingly, the steady-state levels of MEP-1 protein are reduced in all of these mutant backgrounds, suggesting that initial processing of MEP-1 is important for its stabilization. In addition to evaluating MEP-1 protein levels by western blot and providing full quantitative details, we carefully examine these animals for morphological defects that revealed gonadal defects in these mutant animals (Fig. EV3).

Furthermore, we assessed MEP-1 protein levels in *dpf-3; dpf-4* double, *dpf-3; dpf-5* double and *dpf-3; dpf-4; dpf-5* triple mutant animals. We selected *dpf-4* and *dpf-5* because we have observed synthetic sterility in these double mutants (unpublished data). As shown in Fig. 4C,D, deletion of *dpf-5*, but not *dpf-4*, in the *dpf-3* deletion background further reduces MEP-1 levels, suggesting that DPF-3 and DPF-5 likely act in coordination during the processing of nascent MEP-1. In contrast, deletion of *dpf-4* results in a modest increase in the steady-state levels of MEP-1; however, the basis for this effect is currently unclear and will be the subject of future investigation.

5) Fig 5B-E: the images in my PDF file are not of good enough quality to judge the exact defects that may be present.

We apologize for the inconvenience caused by the low image quality in the submitted PDF. We have provided all images in high resolution to allow clear assessment of the observed phenotypes.

Reviewer #1 (Significance (Required)):

Aygün et al. report on the identification of novel in vivo substrates of the peptidase DPF-3 in C. elegans/ DPF-3 is a member of the DPPIV family of di-peptidases with clinical relevance, and a better understanding of the activities in vivo is clearly important. Notably, the authors here show that in vivo DPF-3 can cleave following a threonine, an activity that thus far had only been shown in vitro. In particular, they show and validate this for the protein MEP-1. Loss of DPF-3 leads to a loss of steady-state levels of MEP-1, something that was also found in a mep-1 mutant background that produced MEP-1(T3E) protein that is resistant to DPF-3. This mep-1 allele also recapitulated some of the dpf-3 mutant phenotypes, suggesting that MEP-1 processing, or rather the lack thereof, contributes to the dpf-3 mutant phenotype.

Once again, we thank the reviewer for critically evaluating our manuscript, recognizing the novelty of our work, and providing constructive comments that helped us further improve the

manuscript.

Reviewer #2 (Evidence, reproducibility and clarity (Required)):

Aygun et al. use a mass spectrometry-based assay in C. elegans to identify substrates of the Dipeptidyl Peptidase IV (DPPIV) family member DPF-3. This was motivated by their prior work that showed DPF-3 could cleave after more diverse amino acids than previously thought. After verifying their approach identified genuine DPF-3 targets using an in vitro synthetic peptide and recombinant DPF-3 assay, they focused on MEP-1 which has a non-canonical amino acid cleavage site. They demonstrated that DPF-3 cleavage impacts MEP-1 stability and showed the physiological consequence of disrupting DPF-3 cleavage of MEP-1 is low penetrant reproductive defects and germline defects.

Major Comments:

1. Quantification of western blots with statistics for replicates should be added for Figure 4A.

We apologize for the omission of quantification in Figure 4A. In the revised version, we included quantitative analysis of the western blots with statistical evaluation of biological replicates. In addition, as suggested by Reviewer #1 (comment 3), we included additional samples to rule out background signal and provided a new, fully quantified western blot (Fig. 4A,B).

2. The authors see that the flag::mep-1(T3E) allele shows near complete loss of MEP-1 levels but that MEP-1 levels are not as dramatically changed in dpf-3Δ; flag::mep-1 animals and suggest that this means DPF-3 processing removes a degron signal at the N-terminus of MEP-1 required for the protein stability. Further they argue that the dramatic effect is not observed in dpf-3Δ; flag::mep-1 animals due to the fact that other DPPIV family members could maybe act redundantly to cleave the T3 site in mep-1. However, there currently is no evidence that other DPPIV family members could recognize the T3 site in mep-1 and begs the question of whether the flag tag and T3E mutation elicits some other fate that blocks translation of the mep-1(T3E) mRNA or leads to its degradation. To support that other DPPIV family members act redundantly in the dpf-3Δ; flag::mep-1 animals, the authors could perform the in vitro synthetic peptide assay with other recombinant DPPIV family members to show they are able to cleave MEP-1 in the absence of DPF-3.

We appreciate the reviewer's insightful comments. The reviewer raises a valid concern regarding whether the T3E mutation itself could lead to degradation of the MEP-1 protein. This issue was also noted by Reviewer #1 (comment 4), and we addressed it by generating the following additional mutant transgenic animals: *mep-1(T3G), mep-1(T3M), mep-1(T3L)* and *mep-1(T3I)* and assessed MEP-1 protein levels (Fig. 4A,B), revealing a reduction across all mutant backgrounds. In addition to evaluating MEP-1 protein levels by western blot and providing full quantitative details, we carefully examined these animals for morphological defects that revealed gonadal migration defect in these mutant animals (Fig. EV3) suggesting that initial processing of MEP-1 is important for its function.

Regarding the possibility that other DPPIV family members may act redundantly to process MEP-1, we agree that direct biochemical evidence would be ideal. The *C. elegans* DPPIV family consists of six predicted catalytically active members (based on conservation of the catalytic triad) and one potentially inactive paralog. However, these proteases are not commercially available, and to date, we have only successfully produced recombinant DPF-3 in insect cells, a process that required more than two years of optimization due to severe expression and aggregation issues. Since production of additional recombinant family members seemed thus outside the scope of a revision, we tested our hypothesis instead genetically by examining MEP-1 levels in *dpf-4* or *dpf-5* single mutants, as well as in double or triple mutant backgrounds. We selected *dpf-4* and *dpf-5* because we have observed synthetic sterility in these double mutants (unpublished data). As shown in Fig. 4C,D, deletion of *dpf-5*, but not *dpf-4*, in the *dpf-3* deletion background further reduces MEP-1 levels, suggesting that DPF-3 and DPF-5 likely act in coordination during the processing of nascent MEP-1. In contrast, deletion of *dpf-4* results in a modest increase in the steady-state levels of MEP-1; however, the basis for this effect is currently unclear and will be the subject of future investigation.

3. Why did the authors perform their reproductive assays at 25ºC, which is a heat stress condition? Were the western blots also performed at 25ºC? Does their mep-1(T3E) allele not recapitulate mep-1(q660) phenotypes at 20ºC, which is the standard growth temperature of C. elegans? Would this suggest the role of DPF-3 in regulating MEP-1 is critical for during heat stress and not necessarily during permissive conditions? An explanation for the choice of 25 ºC would be helpful for the reader, and potentially complementary experiments performed at 20 ºC.

We thank the reviewer for this important comment. All experiments in this study, including the TAILS-based substrate identification and western blot analyses, were performed using animals cultured at 25℃. We chose this temperature for consistency, as the TAILS samples were prepared from animals grown at 25℃, and all subsequent experiments were conducted under the same conditions to ensure comparability.

We agree that examining these phenotypes at 20℃ could provide valuable additional insight. As suggested, we repeated the relevant experiments at 20℃ to determine whether the observed effects are specific to heat stress conditions or reflect a more general role of DPF-3 in regulating MEP-1 stability and its function. The *mep-1(T3E)* mutant animals showed a significant reduction in brood size at 20℃, demonstrating that the effect of DPF-3 on MEP-1 stability and function is not temperature dependent and reflects a broader role for DPF-3 in reproductive fitness. We incorporate this data in the revised manuscript (Fig. 5B).

4. Does the DPF-3 processing impact localization of MEP-1?

We thank the reviewer for raising this point, which we were also questioning. We generated transgenic animals carrying a GFP tag inserted at the endogenous mep-1 locus. The GFP epitope was introduced at the same position as the FLAG tag (between amino acids 52 and 53). While we successfully achieved precise integration of the GFP tag, we were unable to obtain viable homozygous lines. Despite repeated attempts, homozygous animals failed to produce progeny, and even heterozygous animals exhibited poor health.

Due to these limitations, we were unable to proceed with crossing these strains into a *dpf-3* null background to directly assess the impact on MEP-1 localization. Nevertheless, to document our efforts, we are providing a “reviewer figure” (GFP-MEP-1 tagging) that includes: (i) western blot analysis demonstrating expression of the full-length GFP::MEP-1 fusion protein, and (ii) representative images of animals carrying the GFP-tagged allele.

We hope that these data demonstrate our sincere efforts to address this important question and will be appreciated by the reviewer.

5. OPTIONAL: The authors propose the role of DPF-3 processing is to fine-tune abundance of MEP-1 and it's interaction complexes, if this model is correct, there could be potential gradations in changes of the chromatin landscape between wild-type, dpf-3Δ; flag::mep-1, and flag::mep-1(T3E) animals that reflective the levels of stable MEP-1 protein.

We thank the reviewer for this insightful suggestion. Assessing potential changes in the chromatin landscape among wild-type, *dpf-3Δ; flag::mep-1*, and *flag::mep-1(T3E)* animals could indeed provide valuable information about the impact of DPF-3 mediated processing on MEP-1 function and its associated complexes. However, such experiments would represent a major undertaking, requiring genome-wide chromatin analyses, and we believe that this is beyond the scope of the current manuscript.

Minor Comments:
1. The fourth paragraph of the results, discussing factors that impact patterns of substrate/product detection was a bit confusing as it is written and where it is placed. The relevance of the different patterns comes later in the MEP-1 results, so it may improve readability to move some of this discussion there.

We thank the reviewer for this helpful suggestion. We moved the text accordingly to incorporate the recommended changes in the revised manuscript.

2. It is unclear why the authors picked GDPH-1 and FAT-1 as candidates to validate the DPF-3 substrates/products found in their screen as these didn't exhibit substrate/product pairs, so some explanation of their choice would be helpful.

Our goal was to validate the TAILS results using an orthogonal approach - an *in vitro* assay with synthetic peptides representing the N-termini of the identified substrates. We selected GPDH-1 and FAT-1 as representative examples because these peptides contained Pro and Ala residues respectively, at the P1 position. These candidates were chosen randomly from the list of putative substrates for initial validation. We included this information in the revised manuscript.

3. On Figure 1B, it could be helpful to label the quadrants of the plot as "DPF-3 substrates" and "DPF-3 products".

Thank you for the suggestion; we labelled the quadrants as suggested in the revised manuscript.

4. It would be helpful to have a graphic with percentages of reproductive phenotypes for the wild-type and mep-1(T3E) animals in Figure 5.

We thank the reviewer for this suggestion and include the suggested graph in the revised manuscript (Fig. 5G).

Reviewer #2 (Significance (Required)):

The work by Aygun et al. is of broad interest and addresses open questions in basic research about post-translational regulation by Dipeptidyl Peptidase IV (DPPIV) family members. The identification of novel substrates and non-canonical cleavage sites is of significance as it challenges current perceptions of DPF-3 targets and advances our understanding of how to study these proteases. Further the authors demonstrate a functional role of DPF-3 in reproduction and through the cleavage of one newly identified target. A strength of the study is the unbiased approach of identifying DPF-3 targets that enabled them to find products cleaved at non-canonical amino acids, but a limitation is the reagents used for lysate preparation; however they acknowledge this. It is another strength that they were able to show a physiological consequence of disrupting the DPF-3 processing of this unexpected target and start to address potential function, although a few additional experiments could bolster their arguments towards this functional role. A limitation in the interpretation of their reproductive assays I see is the choice of growth temperature for the reproductive assays (25ºC) as heat stress can already trigger low penetrance reproductive phenotypes. Further, this entangles their interpretation of the role of DPF-3 processing with its role during stress which could differ from its role during non-stress conditions. My expertise: epigenetics, reproduction, homeostasis, C. elegans

We sincerely thank the reviewer for the thoughtful and constructive evaluation of our work. We appreciate their recognition of the novelty and significance of identifying non-canonical DPF-3 cleavage sites and newly uncovered substrates, as well as the importance of our unbiased proteomic approach. We are also grateful for the reviewer's acknowledgement of the physiological relevance and broad interest of our findings and the strengths of our functional analyses.

Reviewer #3 (Evidence, reproducibility and clarity (Required)):

This is a solid and well-executed study that convincingly identifies DPF-3 as a protease required for MEP-1 processing and stabilization in C. elegans, and explores the molecular and physiological consequences of disrupting this pathway. The combination of biochemical, proteomic, and genetic approaches provides a coherent mechanistic narrative, and multiple lines of evidence support most conclusions. The presentation is generally clear, the data are well described, and the manuscript is carefully organized.

We thank the reviewer for their thoughtful and positive evaluation of our work. We appreciate their recognition of the study's clarity and our integrated biochemical, proteomic, and genetic approaches supporting a coherent framework for DPF-3 mediated MEP-1 processing. We are pleased that the strength and physiological relevance of our findings were clearly conveyed.

1. My main concern relates to the IP-mass spectrometry analyses (Fig. 4 and Supp. Fig. 2), which underpin the claim that MEP-1 complex composition is unchanged in the dpf-3 mutant. The p-values for enriched interactors are quite modest, and it is unclear whether this reflects insufficient biological replicates, technical variability, or high background. The authors should clarify the number of replicates, report replicate correlations and missing-value rates, and use a consistent statistical workflow across comparisons. It would also be helpful to state whether the "match-between-runs" method was used. While the conclusion that complex composition remains unchanged may be correct, the data as presented do not rule out the possibility that the apparent similarity arises from limited statistical power. If possible, targeted validation (e.g. co-IP for key partners such as LET-418 or HDA-1) would strengthen this claim, even though co-IPs do not provide a quantitative view

We thank the reviewer for these detailed and constructive comments, which have meaningfully improved the clarity and rigor of our IP-MS analysis. All IP-MS experiments were performed with three biological replicates per condition, which is now explicitly stated in both the figure legend and the Methods section. To assess technical variability, we computed pairwise Pearson correlations between all samples based on log2 LFQ intensities; biological replicates cluster tightly together, demonstrating high reproducibility across the dataset (please see the figure below). Differential analysis was performed using limma, an empirical Bayes framework that provides regularized variance estimation and robust p-value stabilization even in low-replicate settings with this refined analysis we obtained compelling p-values across all comparisons. MaxQuant processing was performed with Match-Between-Runs (MBR) enabled using the default 0.4-minute matching window; the resulting missing value structure is visualized in the data matrix (please see below, the white values correspond to missing values, and the grey ones to observed intensities), with rates falling within the expected range for IP-MS data of this complexity, and remaining missing values were imputed using MinProb (global) via the einprot pipeline. To further validate that MBR did not introduce artifactual similarities between conditions, we repeated the full analysis with MBR disabled, and the conclusion of unchanged MEP-1 interactome composition in the *dpf-3* mutant background was fully maintained (please see the plots blow).
We appreciate the suggestion for targeted co-immunoprecipitation of key interactors such as LET-418 or HDA-1, and acknowledge that such experiments would provide complementary evidence. Nevertheless, the high degree of correlation between biological replicates, the compelling p-values obtained through our refined limma-based analysis, and the robustness of our conclusions in the absence of MBR collectively indicate that our dataset is sufficiently powered to support the claim of unchanged MEP-1 complex composition in the *dpf-3* mutant background.

Pearson correlations Plot

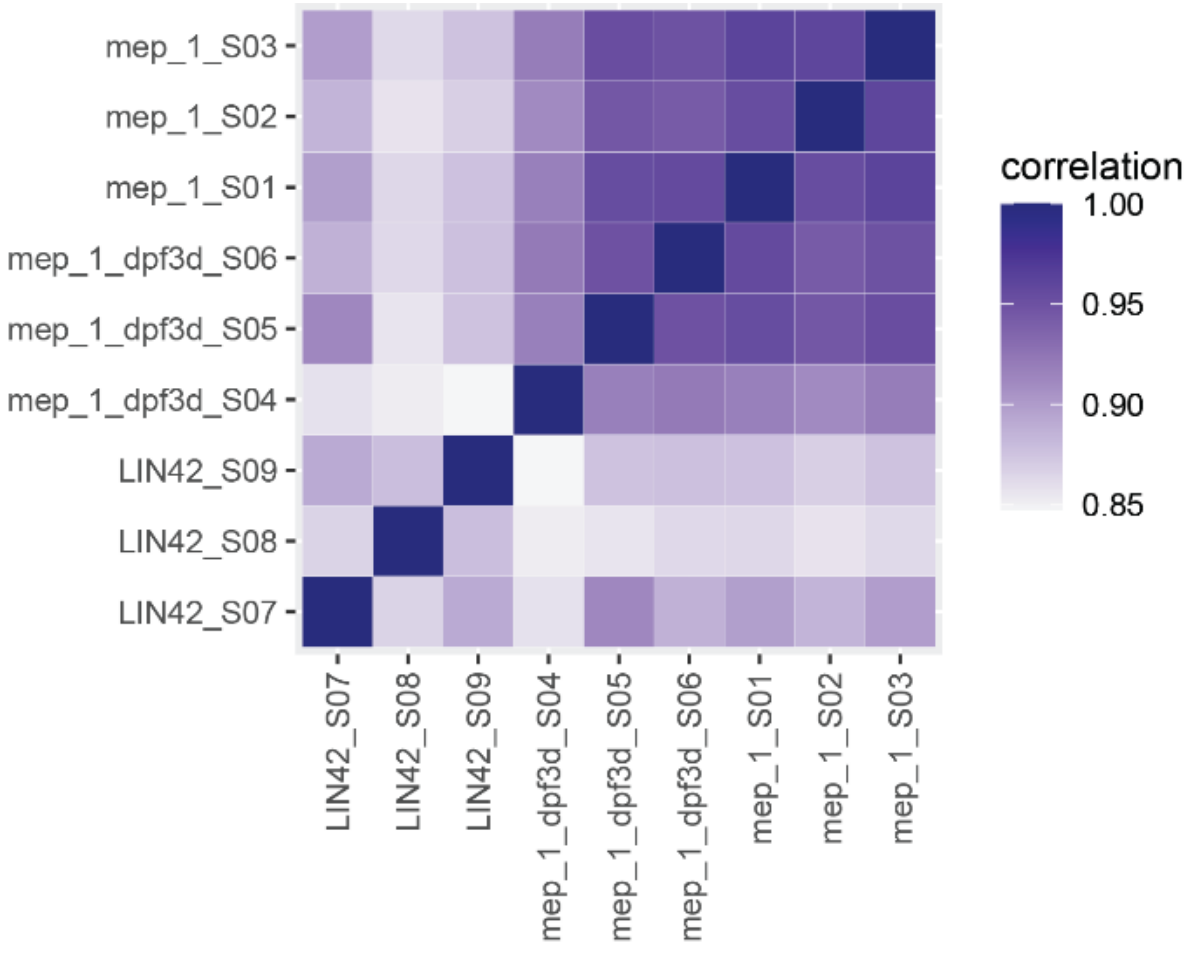

Missing value structure in the data matrix

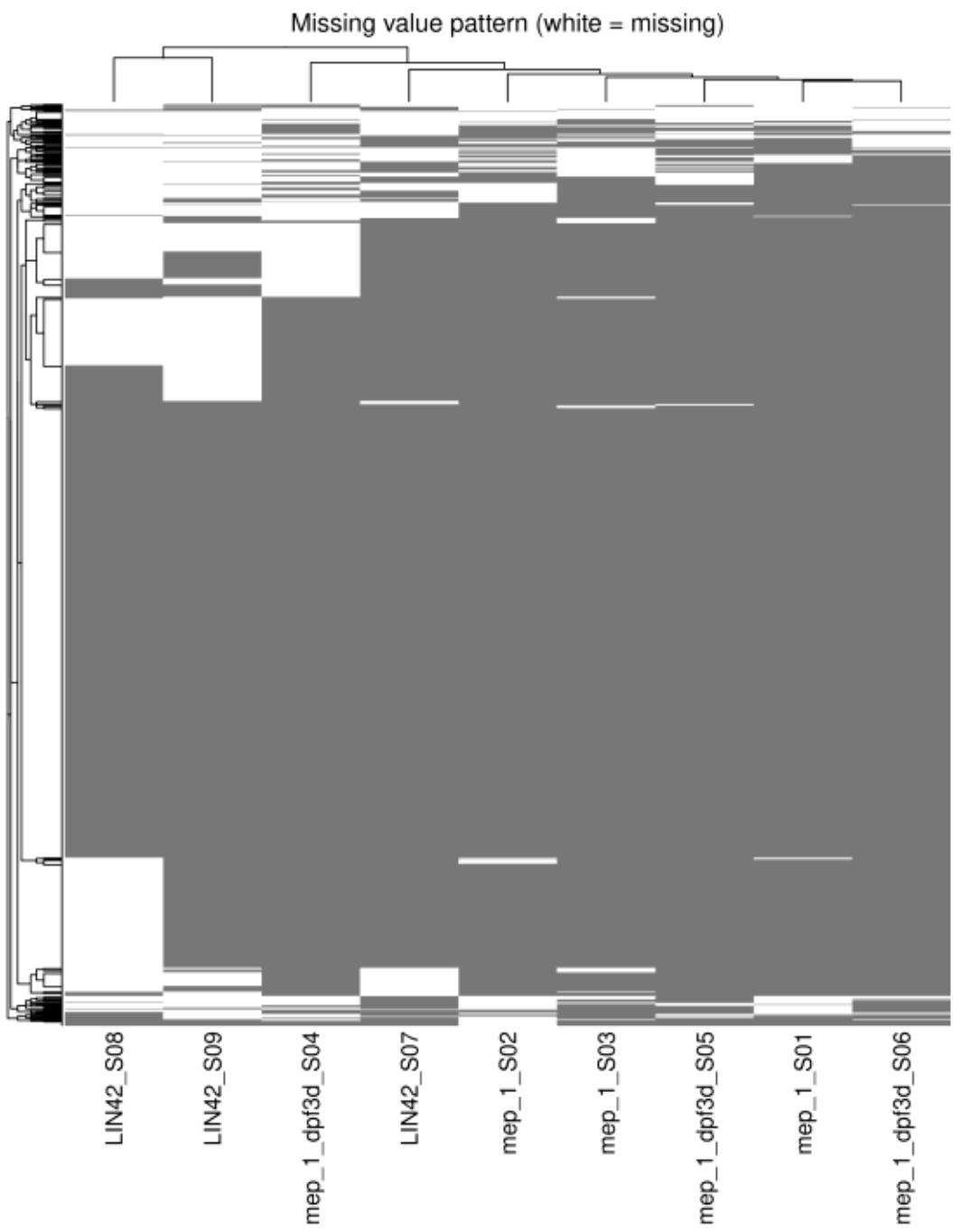

IP-MS analysis of MEP-1 with Match-Between-Runs disabled

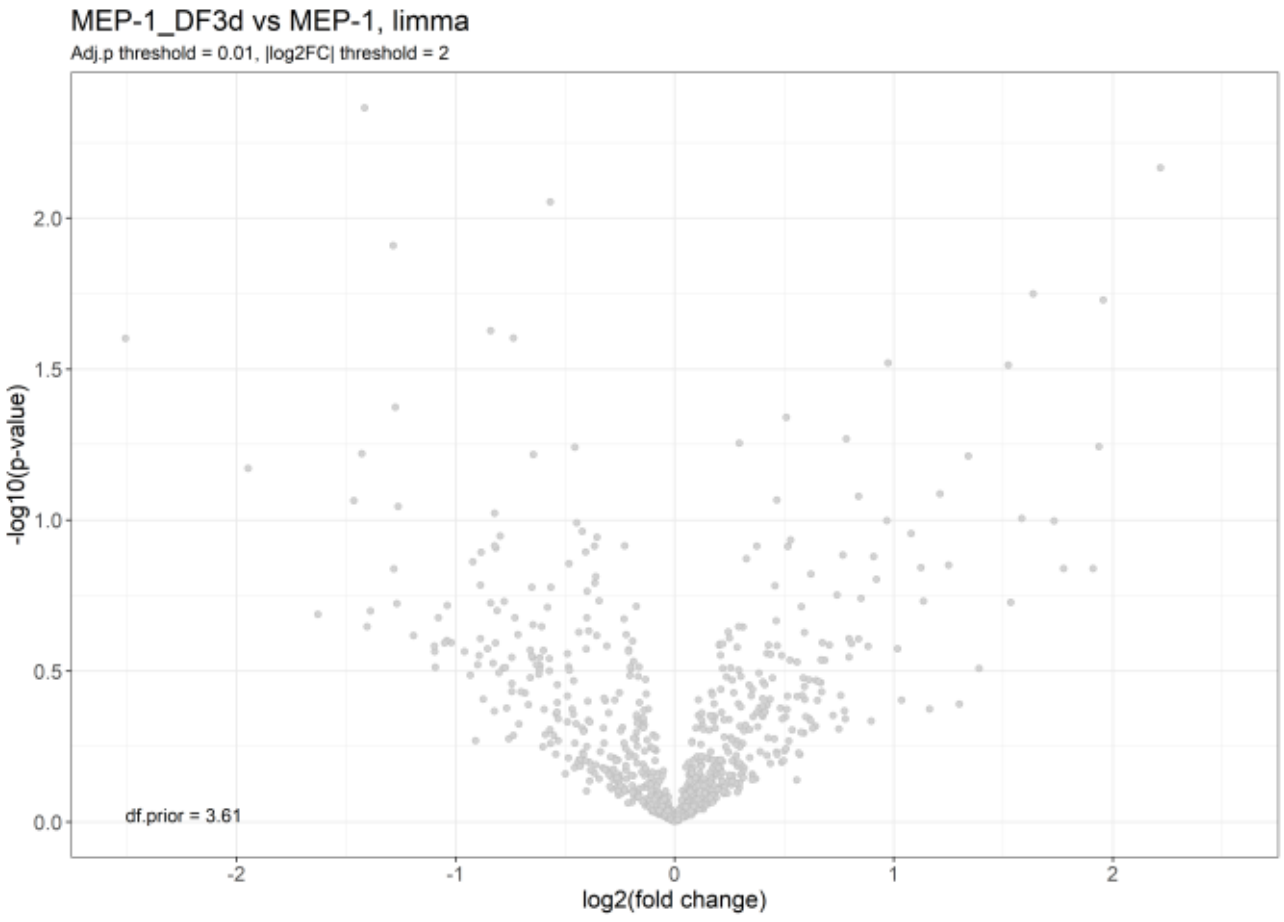

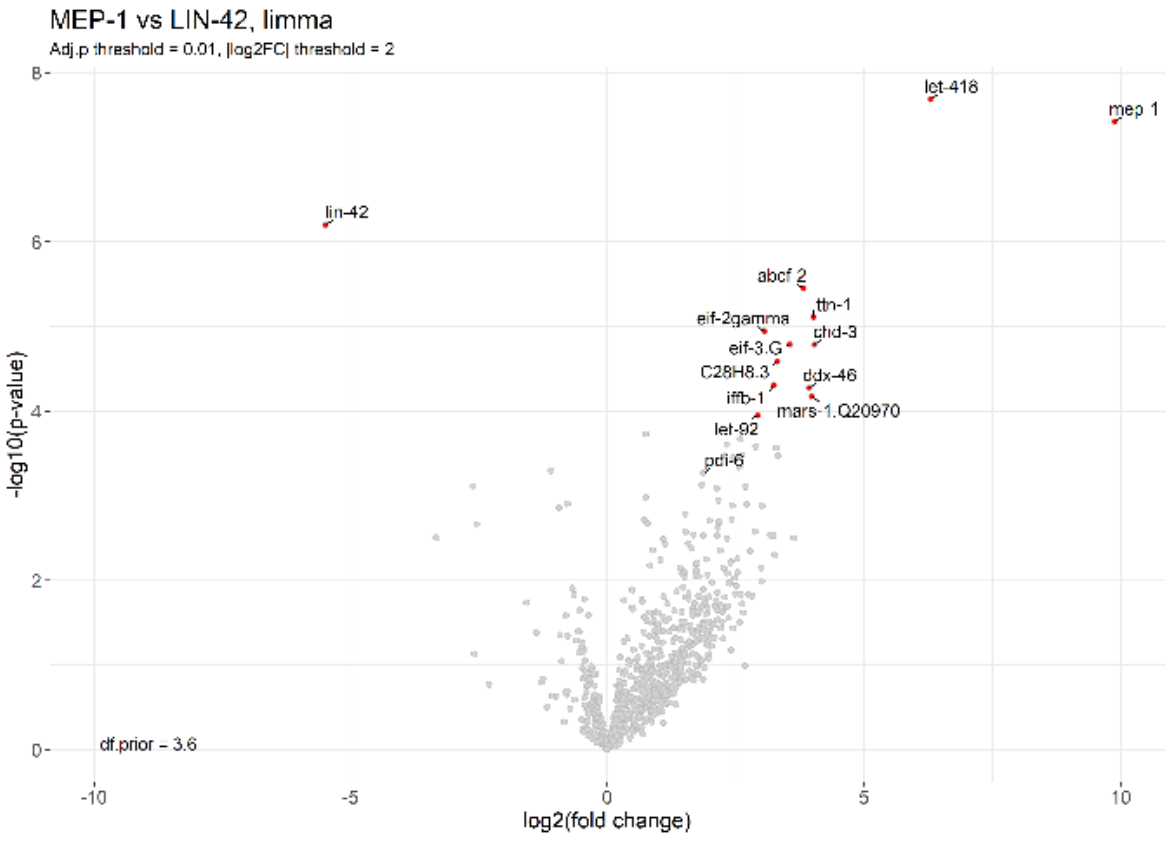

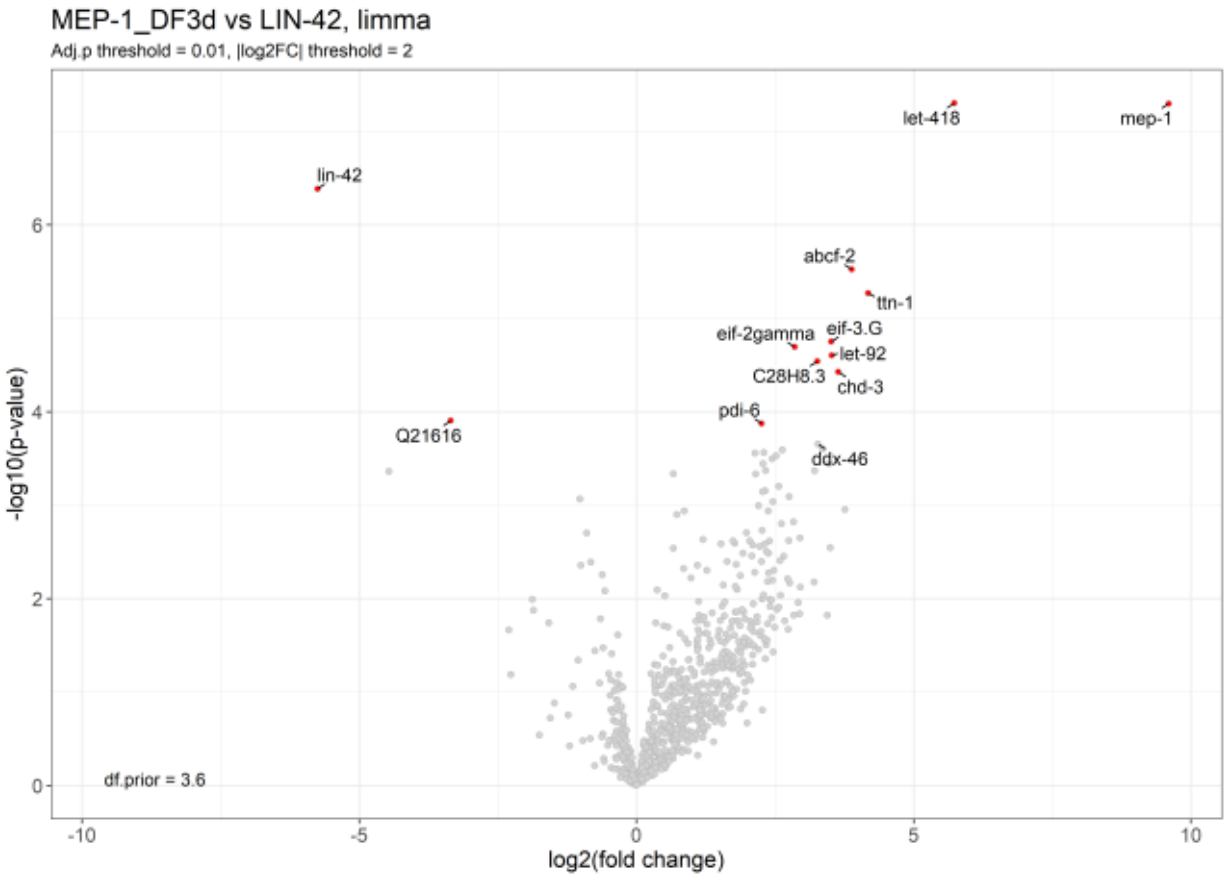

2. Another point concerns the brood-size assays, which were performed at 25 {degree sign}C. This temperature reduces fertility even in wild-type animals, complicating the interpretation of genotype-specific effects. It would be important to report brood size at 20 {degree sign}C and discuss whether the observed fertility defects are temperature-dependent or intrinsic to the mutations.

We thank the reviewer for this valuable comment, which was also raised by reviewer #2 (comment 3). We agree that examining brood size at 20℃ would help clarify whether the observed fertility defects are temperature dependent or intrinsic to the mutations. We quantified the brood size at 20℃. The mutant animals showed a significant reduction in brood

size at 20℃, demonstrating that the effect of DPF-3 on MEP-1 stability and function is not temperature dependent and reflects a broader role for DPF-3 in reproductive fitness. We incorporated this data in the revised manuscript (Fig. 5B).

3. Finally, the authors propose that other DPF family members may compensate for loss of dpf-3, based on the more pronounced destabilization of MEP-1(T3E). This is an interesting idea that could be tested by a targeted RNAi screen or double/triple mutant analysis of other dpf genes in the dpf-3 background.

We thank the reviewer for this insightful comment, which is also in line with comment 2 from reviewer #2. We tested our hypothesis genetically by examining MEP-1 levels in *dpf-4* or *dpf-5* single mutants, as well as in double or triple mutant backgrounds. We selected *dpf-4* and *dpf-5* because we have observed synthetic sterility in these double mutants (unpublished data). We assessed MEP-1 protein levels in *dpf-3; dpf-4* double, *dpf-3; dpf-5* double and *dpf-3; dpf-4; dpf-5* triple mutant animals (Fig. 4C,D). Deletion of *dpf-5*, but not *dpf-4*, in the *dpf-3* deletion background further reduces MEP-1 levels, suggesting that DPF-3 and DPF-5 likely act in coordination during the processing of nascent MEP-1. In contrast, deletion of *dpf-4* results in a modest increase in the steady-state levels of MEP-1; however, the basis for this effect is currently unclear and will be the subject of future investigation.

4. Overall, the experiments are carefully performed and described, but clarification of the proteomic statistics and additional fertility assays would increase confidence in the robustness of the conclusions.

We thank the reviewer for the positive feedback. We have performed additional fertility assays as suggested at 20℃ that strengthens the robustness of our conclusions.
We included the relevant statistical analyses for the proteomics data (as outlined above in response to comment 1).
Reviewer #3 (Significance (Required)):

This study provides an important mechanistic advance in understanding how the protease DPF-3 regulates the stability and function of the chromatin factor MEP-1 in C. elegans. Previous work had implicated DPF-3 in RNA silencing and proteostasis, but its direct substrates and developmental roles were not defined. Using a proteome-wide N-terminomics (TAILS) approach, the authors systematically identify DPF-3 cleavage targets in developing nematodes. This analysis not only recovers canonical DPPIV substrates with Pro or Ala at the P1 position but also uncovers MEP-1 as a noncanonical substrate containing Thr at P1, whose cleavage is essential for its stability and function in germline development.

By integrating quantitative proteomics, mutagenesis, and genetic analysis, the study reveals that loss of DPF-3-dependent cleavage leads to destabilization of MEP-1, defective gonadal migration, and reduced fertility. This discovery broadens the known substrate specificity and biological repertoire of DPF-3, positioning it as a protease that acts on chromatin-associated proteins rather than exclusively on cytosolic or silencing factors. The identification of MEP-1 as a noncanonical target provides a striking example of how proteolytic processing can fine-tune chromatin regulator function in vivo.

The main limitation lies in the modest statistical support for unchanged complex composition in the dpf-3 mutant IP-MS data, which somewhat weakens the conclusion that DPF-3 does not alter MEP-1 complex assembly. Nonetheless, the study presents a coherent and well-supported mechanistic model that significantly extends the functional landscape of DPF-3. The findings will be of broad interest to researchers studying protease biology, chromatin regulation, and germline development, and may have implications for understanding noncanonical DPPIV functions across species.

We thank the reviewer for broadly endorsing our manuscript and for highlighting the significance of our findings. We appreciate the comment regarding the statistical analysis of the MEP-1 protein complex. We provided a more detailed analysis of the immunoprecipitation - mass spectrometry (IP - MS) datasets, including appropriate statistical evaluation, to strengthen the support for our conclusions.

1st Revision - Editorial Decision — 20th May 2026

Dear Dr. Gudipati

Thank you for the submission of your revised manuscript to EMBO reports. I apologise for the delay in getting back to you. There has been a large number of recent submissions. We have now received the full set of referee reports that is copied below and have completed editorial checks.

As you will see, all referees are very positive about the study and request only minor changes to clarify text and figures.

From the editorial side, there are also a few things that we need before we can proceed with the official acceptance of your study:

- Please provide a new email address for Daniel Hess (who no longer works at FMI).

- Please remove the Expanded data information section from the manuscript file.

- Please remove the figures from the manuscript files and provde the legends as plain text after the References at the end of the manuscript.

- Please move the Data Availability Statement before the Acknowledgements.

- Please rename the "Materials and methods" section to Methods.

- Please include statements on sample size calculation and blinding even if no sample size calculation or blinding were done.

- Please rename the "Competing interests" statement to Disclosure and Competing Interests Statement.

- If affiliation #3 is a company, please make a statement related to this in the Disclosure and Competing Interests Statement.

- Please remove the instructions and the example table from the Reagents and Tools Table. Consider moving the primer, C. elegans strain, and antibody information from the individual tables to the Reagents and Tools table.

- CRediT has replaced the traditional author contributions section because it offers a systematic machine readable author contributions format that allows for more effective research assessment. Please remove the Author Contributions section from the manuscript and use the free text boxes beneath each contributing author's name in our online systems to add specific details on the author's contribution. More information is available in our guide to authors.

- Please adjust the format of the reference list and of the in-text citations according to EMBO Journal format (up to 10 author names in the reference list before et al). Please refer to our Guide to Authors for additional information on EMBO J reference format.

- Please double-check to make sure to all relevant funding information in the manuscript is also entered into our submission system. (Missing in the system currently: FENG.02.01-IP.05-M038/25, TREAT-EXP, the Novartis Research Foundation through the Friedrich Miescher Institute for Biomedical Research).

- Please rename the uploaded datasets in the source files, system, and in the manuscript to Dataset EV1 -EV4 and provide each legend as a separate sheet/tab in each Excel file.

- Please note that EMBO press papers are accompanied online by:
A) a short (2 sentences) summary of the findings and their significance,
B) 2-5 short bullet points highlighting the key results, and
C) a synopsis image in .jpg or .png format that is exactly 550 pixels wide and 300 pixels high. Please note that the text needs to be legible at the final size. Please upload this information along with your revised manuscript (the text for A and B should be provided in one separate Word file uploaded as Cover Art/Synopsis Image).

- Please loosen the cropping around the western blots so that there is more space around the bands.

- Can you explain the duplicated cells in the source data for figures 2A, B, C, 3A, B (excel files attached).

- Please doublecheck that individual data points are depicted for all figures with n<5. In particular, check Fig 5G.

- Our production/data editors have asked you to clarify several points in the figure legends (see below). Please incorporate these changes in the manuscript and return the revised file with tracked changes with your final manuscript submission.

- Please indicate the statistical test used for data analysis in the legends of figures 3D, 4B, D, E, F; 5A, B, H;EV2 B, D.
- Please note that information related to n is missing in the legends of figures 4E, F

Sincerely,
Kurt Weir
Editor
EMBO Reports

Referee #1:

The authors have addressed our concerns and improved their manuscript. We support its publication, but there are two minor issues should be addressed. The newly added western blots are too narrowly cropped; the authors should leave some space above and below the band of interest (even if uncropped images will need to be provided anyway). The second issue is that one of the western blots in Fig. 4c is missing annotation labels.

Referee #2:

The authors have addressed all of the concerns of this reviewer, and the other reviewers. The additional data and clarifications have further strengthened this already strong, and significant study.

Dear Dr Weir,

Thank you once again for guiding us through the manuscript submission. Please see below for point by point responses (in blue color). We will be happy to provide any other missing/additional information as required.

Best Regards

Rajani

- Please provide a new email address for Daniel Hess (who no longer works at FMI).

Here is his private e-mail id. 1961danielhess@gmail.com

- Please remove the Expanded data information section from the manuscript file.

Removed as suggested

- Please remove the figures from the manuscript files and provde the legends as plain text after the References at the end of the manuscript.

Provided as suggested

- Please move the Data Availability Statement before the Acknowledgements.

Done

- Please rename the "Materials and methods" section to Methods.

Done

- Please include statements on sample size calculation and blinding even if no sample size calculation or blinding were done.

No sample size calculation or blinding was done. Should we include this statement in the manuscript? If so, could you kindly guide us, where in the manuscript we have to mention this.

- Please rename the "Competing interests" statement to Disclosure and Competing Interests Statement.

Done

- If affiliation #3 is a company, please make a statement related to this in the Disclosure and Competing Interests Statement.

This is not a company. The Swiss Institute of Bioinformatics is an academic and not for profit foundation.

- Please remove the instructions and the example table from the Reagents and Tools Table. Consider moving the primer, C. elegans strain, and antibody information from the individual tables to the Reagents and Tools table.

Thank you for indicating. We have removed the instructions and the example table as suggested. We have now moved the Primers, C. elegans strains to the Reagents and Tools table. The antibodies used in this study are already listed in the Reagents and Tools table.

- CRediT has replaced the traditional author contributions section because it offers a systematic machine readable author contributions format that allows for more effective research assessment. Please remove the Author Contributions section from the manuscript and use the free text boxes beneath each contributing author's name in our online systems to add specific details on the author's contribution. More information is available in our guide to authors.

Thank you for pointing out. I have removed the Author Contributions section. Updated the contributions online.

- Please adjust the format of the reference list and of the in-text citations according to EMBO Journal format (up to 10 author names in the reference list before et al). Please refer to our Guide to Authors for additional information on EMBO J reference format.

Adjusted as suggested.

- Please double-check to make sure to all relevant funding information in the manuscript is also entered into our submission system. (Missing in the system currently: FENG.02.01-IP.05-M038/25, TREAT-EXP, the Novartis Research Foundation through the Friedrich Miescher Institute for Biomedical Research).

We have included all the information related to funding. Please note that the "NCCR RNA & Disease" is not popping up in the dropdown menu.

- Please rename the uploaded datasets in the source files, system, and in the manuscript to Dataset EV1 -EV4 and provide each legend as a separate sheet/tab in each Excel file.

Done as suggested.

- Please note that EMBO press papers are accompanied online by:
A) a short (2 sentences) summary of the findings and their significance,
B) 2-5 short bullet points highlighting the key results, and
C) a synopsis image in .jpg or .png format that is exactly 550 pixels wide and 300 pixels high. Please note that the text needs to be legible at the final size. Please upload this information along with your revised manuscript (the text for A and B should be provided in one separate Word file uploaded as Cover Art/Synopsis Image).

We have provided the text for A and B in "Cover Art" file as suggested. We have also uploaded the Synopsis image in .png format.

- Please loosen the cropping around the western blots so that there is more space around the bands.

Thank you for the suggestion and we have changed the blots as suggested.

- Can you explain the duplicated cells in the source data for figures 2A, B, C, 3A, B (excel files attached).

The experiments indicated in these figures are *in vitro* assays in which the peptides were digested with either wild-type (WT) or catalytic-dead DPF-3. The "0" time point corresponds to the stage at which an aliquot was removed before the reaction mixture was split into two tubes and the respective enzymes were added. Therefore, the "0" time point is common to both conditions, which is why identical values are shown.

- Please doublecheck that individual data points are depicted for all figures with n<5. In particular, check Fig 5G.

We have shown the individual data points for all figures with n < 5. With regard to Fig. 5G, the bars represent percentages rather than individual animals. The total number of animals assayed is stated in the text, which we reproduce below for reference.

"Phenotypic defects in the germline were observed in over 70% of mep-1(T3E) mutant animals (n = 50), compared to approximately 15% of wild-type controls (n = 50). In mep-1(T3E) mutant animals, the most common abnormalities included premature oocyte formation (~20%), enlarged oocytes (~30%), and gonadal arm migration defects (~25%) (The percentages refer to all the animals)".

- Our production/data editors have asked you to clarify several points in the figure legends (see below). Please incorporate these changes in the manuscript and return the revised file with tracked changes with your final manuscript submission.

- Please indicate the statistical test used for data analysis in the legends of figures 3D, 4B, D, E, F; 5A, B, H;EV2 B, D.

We have used the following statistical tests. The same is mentioned in the edited legends of the figures.

3D- Unpaired ttest

4B- One-way ANOVA

4D- One-way ANOVA

4E- Limma (already written in the legend)

4F- Limma (already written in the legend)

5A- One-way ANOVA

5B- One-way ANOVA

5H- Unpaired ttest

EV2 B- Unpaired ttest

EV2 D- Limma (already written in the legend)

- Please note that information related to n is missing in the legends of figures 4E, F

Apologies for omitting. We have now included the following statement in the legend of Figures 4E, F. “The average of three biological replicates is indicated”.

Referee #1:

The authors have addressed our concerns and improved their manuscript. We support its publication, but there are two minor issues should be addressed. The newly added western blots are too narrowly cropped; the authors should leave some space above and below the band of interest (even if uncropped images will need to be provided anyway). The second issue is that one of the western blots in Fig. 4c is missing annotation labels.

We thank the reviewer for endorsing our manuscript and apologies for missing the labels. We have included them and also cropped the western blots as suggested and uploaded new figures.

Referee #2:

The authors have addressed all of the concerns of this reviewer, and the other reviewers. The additional data and clarifications have further strengthened this already strong, and significant study.

We thank the reviewer for their time and effort, and for supporting our manuscript.

Rajani Gudipati
Adam Mickiewicz University
Center for Advanced Technologies
Poland

Dear Dr. Gudipati,

I am pleased to inform you that your manuscript has been accepted for publication in EMBO reports. Your manuscript will be processed for publication by EMBO Press. It will be copy edited and you will receive page proofs prior to publication. Please note that you will be contacted by Springer Nature Author Services to complete licensing and payment information.

You may qualify for financial assistance for your publication charges - either via a Springer Nature fully open access agreement or an EMBO initiative. Check your eligibility: https://link.springer.com/journal/44319/how-to-publish-with-us

Should you be planning a Press Release on your article, please notify the production team via this form in order to coordinate publication and release dates: https://forms.cloud.microsoft/e/eH4A8JVP6g

If you have any questions, please do not hesitate to contact the Editorial Office. Thank you for your contribution to EMBO Reports.

Yours sincerely,

Kurt Weir
Editor
EMBO Reports

>>> Please note that it is EMBO Reports policy for the transcript of the editorial process (containing referee reports and your response letter) to be published as an online supplement to each paper. If you do NOT want this, you will need to inform the Editorial Office via email immediately. More information is available here: https://link.springer.com/partners/embo-press/editorial-policies#Peer%20review

EMBO Press Author Checklist

Corresponding Author Name: Rajani Kanth Gudipati
Journal Submitted to: EMBO reports
Manuscript Number: EMBOR-2025-63385V1-T

USEFUL LINKS FOR COMPLETING THIS FORM
The EMBO Journal - Author Guidelines
EMBO Reports - Author Guidelines
Molecular Systems Biology - Author Guidelines
EMBO Molecular Medicine - Author Guidelines

Reporting Checklist for Life Science Articles (updated January
This checklist is adapted from Materials Design Analysis Reporting (MDAR) Checklist for Authors. MDAR establishes a minimum set of requirements in transparent reporting in the life sciences (see Statement of Task: 10.31222/osf.io/9sm4x). Please follow the journal's guidelines in preparing your
Please note that a copy of this checklist will be published alongside your article.

Abridged guidelines for figures
1. Data
The data shown in figures should satisfy the following conditions:
- the data were obtained and processed according to the field's best practice and are presented to reflect the results of the experiments in an accurate and unbiased manner.
- ideally, figure panels should include only measurements that are directly comparable to each other and obtained with the same assay.
- plots include clearly labeled error bars for independent experiments and sample sizes. Unless justified, error bars should not be shown for technical
- if n<5, the individual data points from each experiment should be plotted. Any statistical test employed should be justified.
- Source Data should be included to report the data underlying figures according to the guidelines set out in the authorship guidelines on Data

2. Captions
Each figure caption should contain the following information, for each panel where they are relevant:
- a specification of the experimental system investigated (eg cell line, species name).
- the assay(s) and method(s) used to carry out the reported observations and measurements.
- an explicit mention of the biological and chemical entity(ies) that are being measured.
- an explicit mention of the biological and chemical entity(ies) that are altered/varied/perturbed in a controlled manner.
- the exact sample size (n) for each experimental group/condition, given as a number, not a range;
- a description of the sample collection allowing the reader to understand whether the samples represent technical or biological replicates (including how many animals, litters, cultures, etc.).
- a statement of how many times the experiment shown was independently replicated in the laboratory.
- definitions of statistical methods and measures:
  - common tests, such as t-test (please specify whether paired vs. unpaired), simple χ2 tests, Wilcoxon and Mann-Whitney tests, can be unambiguously identified by name only, but more complex techniques should be described in the methods section;
  - are tests one-sided or two-sided?
  - are there adjustments for multiple comparisons?
  - exact statistical test results, e.g., P values = x but not P values < x;
  - definition of 'center values' as median or average;
  - definition of error bars as s.d. or s.e.m.

Please complete ALL of the questions below.
Select "Not Applicable" only when the requested information is not relevant for your study.

Materials

Newly Created Materials	Information included in the manuscript?	In which section is the information available? (Reagents and Tools Table, Materials and Methods, Figures, Data Availability Section)
New materials and reagents need to be available; do any restrictions apply?	Yes	Data availability

Antibodies	Information included in the manuscript?	In which section is the information available? (Reagents and Tools Table, Materials and Methods, Figures, Data Availability Section)
For **antibodies** provide the following information:<br>- Commercial antibodies: RRID (if possible) or supplier name, catalogue number and or/clone number<br>- Non-commercial: RRID or citation	Not Applicable	

DNA and RNA sequences	Information included in the manuscript?	In which section is the information available? (Reagents and Tools Table, Materials and Methods, Figures, Data Availability Section)
**Short novel DNA or RNA including primers, probes**: provide the sequences.	Yes	Reagents and Tools Table

Cell materials	Information included in the manuscript?	In which section is the information available? (Reagents and Tools Table, Materials and Methods, Figures, Data Availability Section)
**Cell lines:** Provide species information, strain. Provide accession number in repository **OR** supplier name, catalog number, clone number, and/**OR** RRID.	Not Applicable	
**Primary cultures:** Provide species, strain, sex of origin, genetic modification status.	Not Applicable	
Report if the cell lines were recently **authenticated** (e.g., by STR profiling) and tested for mycoplasma contamination.	Not Applicable	

Experimental animals	Information included in the manuscript?	In which section is the information available? (Reagents and Tools Table, Materials and Methods, Figures, Data Availability Section)
**Laboratory animals or Model organisms:** Provide species, strain, sex, age, genetic modification status. Provide accession number in repository **OR** supplier name, catalog number, clone number, **OR** RRID.	Yes	Reagents and Tools Table
**Animal observed in or captured from the field:** Provide species, sex, and age where possible.	Not Applicable	
Please detail **housing and husbandry conditions**.	Not Applicable	

Plants and microbes	Information included in the manuscript?	In which section is the information available? (Reagents and Tools Table, Materials and Methods, Figures, Data Availability Section)
**Plants:** provide species and strain, ecotype and cultivar where relevant, unique accession number if available, and source (including location for collected wild specimens).	Not Applicable	
**Microbes:** provide species and strain, unique accession number if available, and source.	Not Applicable	

Human research participants	Information included in the manuscript?	In which section is the information available? (Reagents and Tools Table, Materials and Methods, Figures, Data Availability Section)
If collected and within the bounds of privacy constraints report on age, sex and gender or ethnicity for all study participants.	Not Applicable	

Core facilities	Information included in the manuscript?	In which section is the information available? (Reagents and Tools Table, Materials and Methods, Figures, Data Availability Section)
If your work benefited from core facilities, was their service mentioned in the acknowledgments section?	Yes	Acknowledgements

Design

Study protocol	Information included in the manuscript?	In which section is the information available? (Reagents and Tools Table, Materials and Methods, Figures, Data Availability Section)
If study protocol has been **pre-registered, provide DOI in the manuscript**. For clinical trials, provide the trial registration number **OR** cite DOI.	Not Applicable	
Report the **clinical trial registration number** (at ClinicalTrials.gov or equivalent), where applicable.	Not Applicable	

Laboratory protocol	Information included in the manuscript?	In which section is the information available? (Reagents and Tools Table, Materials and Methods, Figures, Data Availability Section)
Provide DOI OR other citation details if **external detailed step-by-step protocols** are available.	Not Applicable	

Experimental study design and statistics	Information included in the manuscript?	In which section is the information available? (Reagents and Tools Table, Materials and Methods, Figures, Data Availability Section)
Include a statement about **sample size** estimate even if no statistical methods were used.	Yes	Methods
Were any steps taken to minimize the effects of subjective bias when allocating animals/samples to treatment (e.g. **randomization procedure**)? If yes, have they been described?	Not Applicable	
Include a statement about **blinding** even if no blinding was done.	Yes	Methods
Describe **inclusion/exclusion criteria** if samples or animals were excluded from the analysis. Were the criteria pre-established?<br><br>If sample or data points were omitted from analysis, report if this was due to attrition or intentional exclusion and provide justification.	Not Applicable	
For every figure, are **statistical tests** justified as appropriate? Do the data meet the assumptions of the tests (e.g., normal distribution)? Describe any methods used to assess it. Is there an estimate of variation within each group of data? Is the variance similar between the groups that are being statistically compared?	Not Applicable	

Sample definition and in-laboratory replication	Information included in the manuscript?	In which section is the information available? (Reagents and Tools Table, Materials and Methods, Figures, Data Availability Section)
In the figure legends: state number of times the experiment was **replicated** in laboratory.	Yes	Results
In the figure legends: define whether data describe **technical or biological replicates**.	Yes	Results

Ethics

Ethics	Information included in the manuscript?	In which section is the information available? (Reagents and Tools Table, Materials and Methods, Figures, Data Availability Section)
Studies involving **human participants**: State details of **authority granting ethics approval** (IRB or equivalent committee(s), provide reference number for approval.	Not Applicable	
Studies involving **human participants**: Include a statement confirming that **informed consent** was obtained from all subjects and that the experiments conformed to the principles set out in the WMA Declaration of Helsinki and the Department of Health and Human Services Belmont Report.	Not Applicable	
Studies involving **human participants:** For publication of **patient photos**, include a statement confirming that consent to publish was obtained.	Not Applicable	
Studies involving experimental **animals**: State details of **authority granting ethics approval** (IRB or equivalent committee(s), provide reference number for approval. Include a statement of compliance with ethical regulations.	Not Applicable	
Studies involving **specimen and field samples:** State if relevant **permits** obtained, provide details of authority approving study; if none were required, explain why.	Not Applicable	

Dual Use Research of Concern (DURC)	Information included in the manuscript?	In which section is the information available? (Reagents and Tools Table, Materials and Methods, Figures, Data Availability Section)
Could your study fall under dual use research restrictions? Please check biosecurity documents and list of **select agents and toxins** (CDC): https://www.selectagents.gov/sat/list.htm	Not Applicable	
If you used a select agent, is the security level of the lab appropriate and reported in the manuscript?	Not Applicable	
If a study is subject to dual use research of concern regulations, is the name of the **authority granting approval and reference number** for the regulatory approval provided in the manuscript?	Not Applicable	

Reporting

The MDAR framework recommends adoption of discipline-specific guidelines, established and endorsed through community initiatives. Journals have their own policy about requiring specific guidelines and recommendations to complement MDAR.

Adherence to community standards	Information included in the manuscript?	In which section is the information available? (Reagents and Tools Table, Materials and Methods, Figures, Data Availability Section)
State if relevant guidelines or checklists (e.g., **ICMJE, MIBBI, ARRIVE, PRISMA**) have been followed or provided.	Not Applicable	
For **tumor marker prognostic studies,** we recommend that you follow the **REMARK** reporting guidelines (see link list at top right). See author guidelines, under 'Reporting Guidelines'. Please confirm you have followed these guidelines.	Not Applicable	
For **phase II and III randomized controlled trials**, please refer to the **CONSORT** flow diagram (see link list at top right) and submit the CONSORT checklist (see link list at top right) with your submission. See author guidelines, under 'Reporting Guidelines'. Please confirm you have submitted this list.	Not Applicable	

Data Availability

Data availability	Information included in the manuscript?	In which section is the information available? (Reagents and Tools Table, Materials and Methods, Figures, Data Availability Section)
Have **primary datasets** been deposited according to the journal's guidelines (see 'Data Deposition' section) and the respective accession numbers provided in the Data Availability Section?	Yes	data availability
Were **human clinical and genomic datasets** deposited in a public access-controlled repository in accordance to ethical obligations to the patients and to the applicable consent agreement?	Not Applicable	
Are **computational models** that are central and integral to a study available without restrictions in a machine-readable form? Were the relevant accession numbers or links provided?	Not Applicable	
If publicly available data were reused, provide the respective **data citations in the reference list**.	Not Applicable	
