# Supplementary material for: Cleavage of MEP-1 by DPF-3 reveals novel substrate specificity and its impact on reproductive fitness
Source: EMBO Rep. 2026 Jun 19;27(15):4524–43. doi: 10.1038/s44319-026-00844-y (PMC13458333; doi:10.1038/s44319-026-00844-y)
Supplement: Supplementary file 2 [file 44319_2026_844_MOESM9_ESM.pdf]

Expanded View Figures

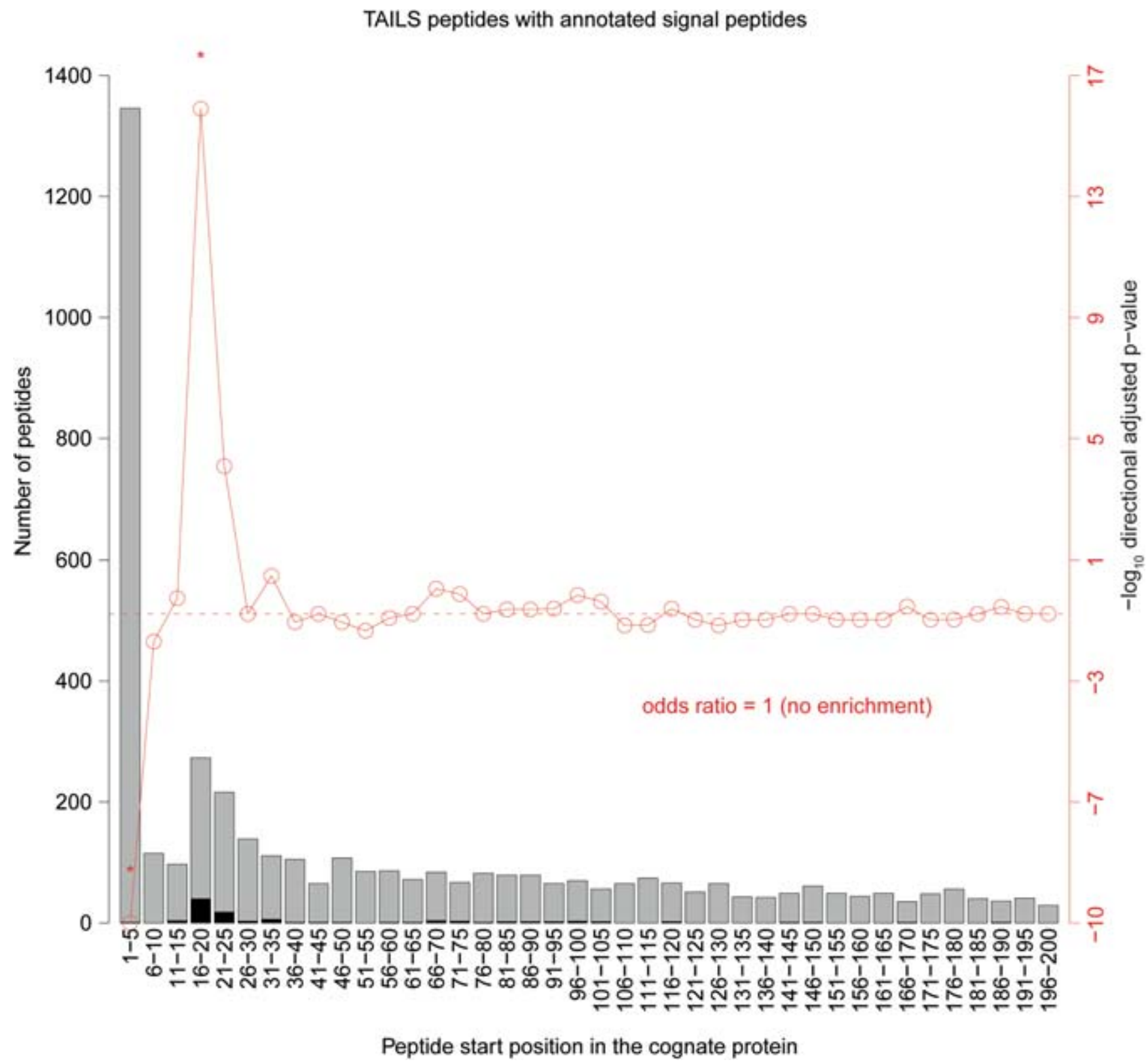

Figure EV1. TAILS enriches protein N-termini.

Starting positions of peptides identified by TAILS are shown in 5 amino acid bins relative to the annotated start site for peptides originating within the first 200 amino acids. Peptides derived from proteins with an annotated signal peptide are indicated in black. The negative log10 directional, adjusted *P* values (Fisher's exact test) testing for the enrichment of signal peptides in proteins at a given bin position are shown in red. Null hypothesis: the fraction of signal peptide annotations is independent of the start position (odds ratio = 1). There is a significant underrepresentation of annotated signal peptides in the TAILS peptide position bins 1–5, and an overrepresentation between 16 and 25 (red asterisks).

 © The Author(s)

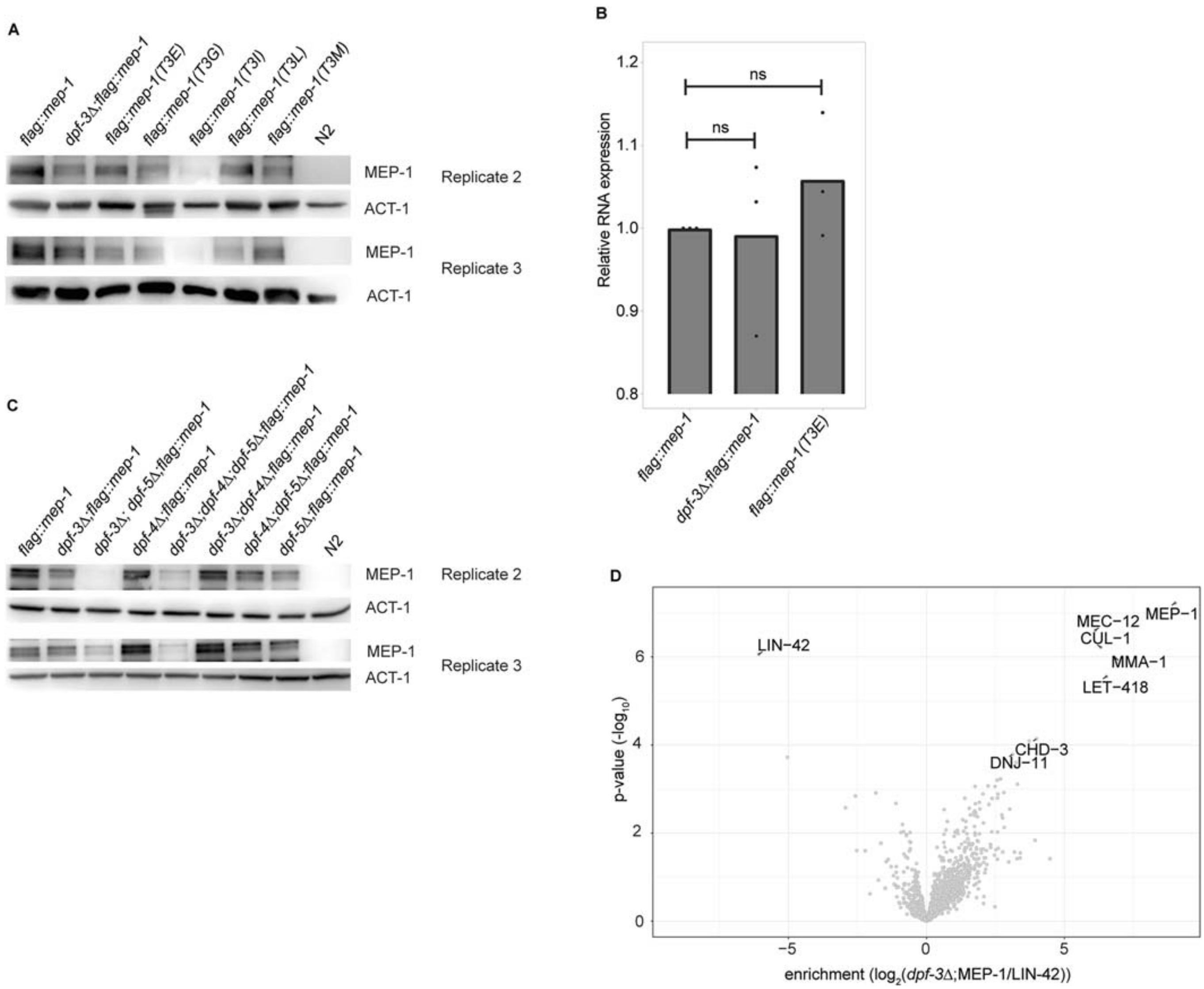

Figure EV2. mep-1 transcript levels are unaffected by dpf-3, and MEP-1 interactors are unchanged in dpf-3Δ mutant animals.

(**A**) Western blot showing reduced MEP-1 levels in dpf-3Δ animals and marked depletion of cleavage-resistant MEP-1(T3) mutant protein. Replicates 2 and 3. (**B**) RT-qPCR quantification of mep-1 mRNA in wild-type, *dpf-3Δ*, and *mep-1(t3e)* mutant animals showing no significant differences in transcript abundance. An unpaired *t* test was used to calculate the *P* value. (**C**) Western blot showing MEP-1 levels in different dpf mutant animals. Replicates 2 and 3. (**D**) IP-MS of MEP-1. The *x* axis indicates the $\log_2$-fold enrichment of the bait protein (MEP-1) and its interacting partners over control (LIN-42). Each dot represents a protein group. The *y* axis of the volcano plot indicates the log10 *P* values that were calculated using limma. An adjusted *P* value threshold of 0.01 was used. Significantly changed ones ($\log_2 > 2$) are colored red, and the rest are indicated in gray. The average of three biological replicates is indicated.

© The Author(s)

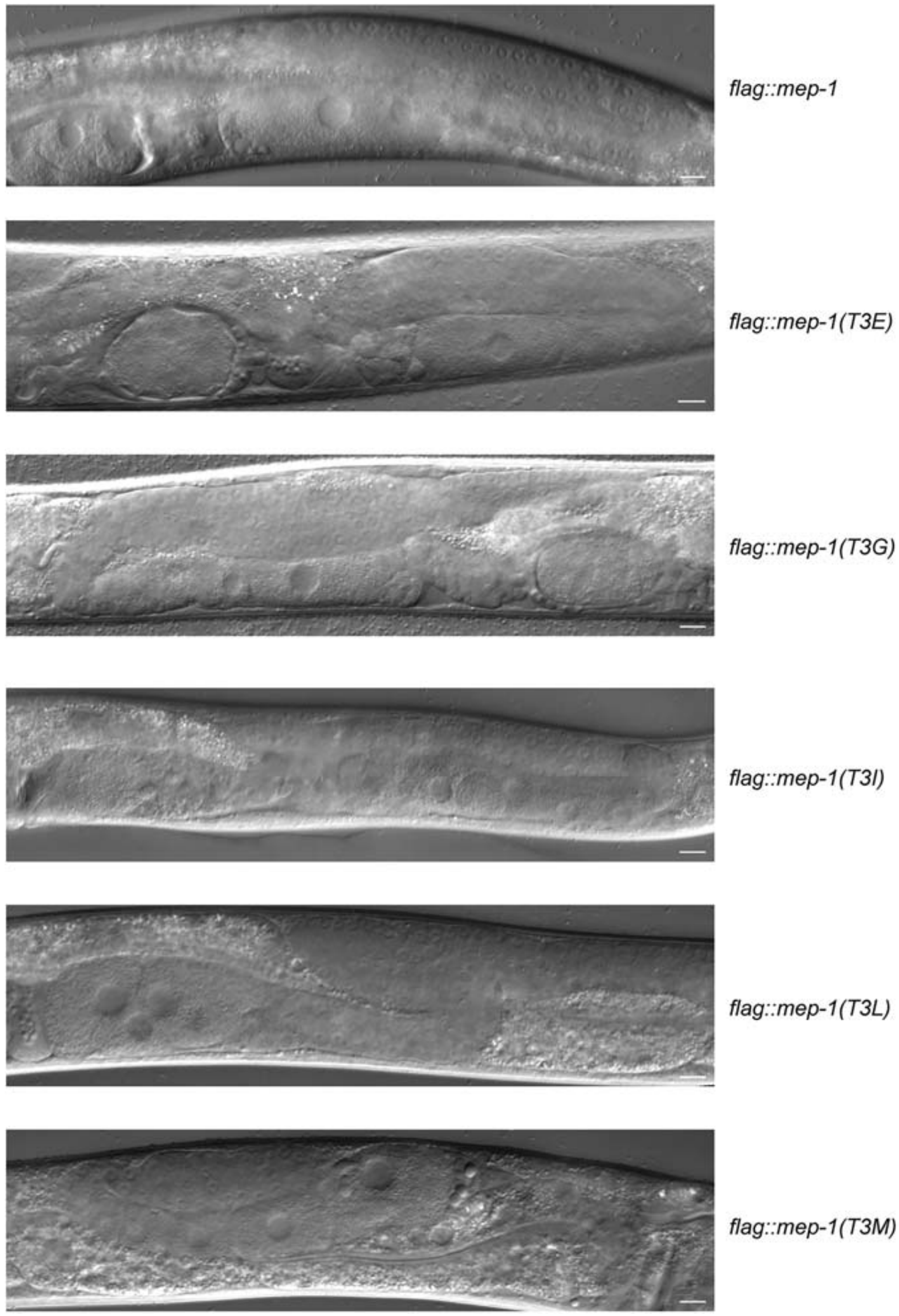

Figure EV3. DPF-3 refractive mep-1 mutant animals show gonadal defects.

Representative Differential Interference Contrast (DIC) images of wild-type and various mep-1(T3) mutant animals grown at 25 °C. All the animals shown here have a 1x *flag* epitope inserted in endogenous *mep-1*.

 © The Author(s)
