# Supplementary material for: Cleavage of MEP-1 by DPF-3 reveals novel substrate specificity and its impact on reproductive fitness
Source: EMBO Rep. 2026 Jun 19;27(15):4524–43. doi: 10.1038/s44319-026-00844-y (PMC13458333; doi:10.1038/s44319-026-00844-y)
Supplement: Supplementary file 3 [file 44319_2026_844_MOESM4_ESM.zip › Fig 1/Fig 1 A/Fig 1A.docx]

Fig. 1A is a cartoon.
