# Supplementary material for: Cleavage of MEP-1 by DPF-3 reveals novel substrate specificity and its impact on reproductive fitness
Source: EMBO Rep. 2026 Jun 19;27(15):4524–43. doi: 10.1038/s44319-026-00844-y (PMC13458333; doi:10.1038/s44319-026-00844-y)
Supplement: Supplementary file 5 [file 44319_2026_844_MOESM6_ESM.zip › Fig 3/Fig 3C/Fig 3C.docx]

Fig. 3C is a cartoon as depicted below.
