# Supplementary material for: Cleavage of MEP-1 by DPF-3 reveals novel substrate specificity and its impact on reproductive fitness
Source: EMBO Rep. 2026 Jun 19;27(15):4524–43. doi: 10.1038/s44319-026-00844-y (PMC13458333; doi:10.1038/s44319-026-00844-y)
Supplement: Supplementary file 5 [file 44319_2026_844_MOESM6_ESM.zip › Fig 3/Fig 3D/Fig. 3D.docx]

Fig. 3D. The bar plot is made from the proteomics data deposited at PRIDE.

<http://www.ebi.ac.uk/pride> (Username: [reviewer_pxd065105@ebi.ac.uk](mailto:reviewer_pxd065105@ebi.ac.uk) Password: HKB6KqnWGHAJ).

The URL is <https://www.ebi.ac.uk/pride/profile/reviewer_pxd065105>.
