# Supplementary material for: Cleavage of MEP-1 by DPF-3 reveals novel substrate specificity and its impact on reproductive fitness
Source: EMBO Rep. 2026 Jun 19;27(15):4524–43. doi: 10.1038/s44319-026-00844-y (PMC13458333; doi:10.1038/s44319-026-00844-y)
Supplement: Supplementary file 6 [file 44319_2026_844_MOESM7_ESM.zip › Fig 4/Fig 4 A/fig 1 A mep-1 actin.pdf]

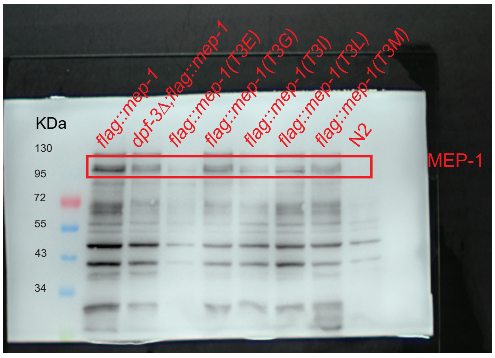
KDa
130
95
72
55
43
34
flag::mep-1
dpf-3Δ;flag::mep-1
flag::mep-1(T3E)
flag::mep-1(T3G)
flag::mep-1(T3I)
flag::mep-1(T3L)
flag::mep-1(T3M)
N2
MEP-1

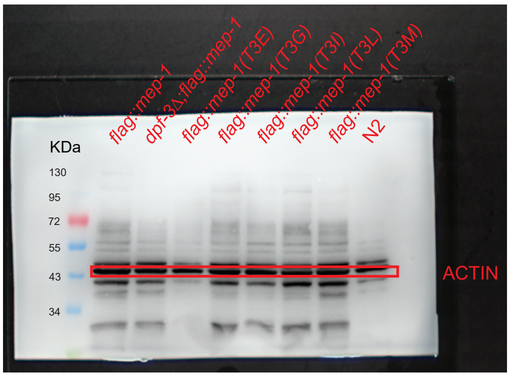
KDa
130
95
72
55
43
34
flag::mep-1
dpf-3Δ;flag::mep-1
flag::mep-1(T3E)
flag::mep-1(T3G)
flag::mep-1(T3I)
flag::mep-1(T3L)
flag::mep-1(T3M)
N2
ACTIN
